# Chutes and ladders: collaborating across disciplines to improve mental and physical healthcare for larger-bodied people

**DOI:** 10.3389/fpsyt.2025.1589858

**Published:** 2025-11-18

**Authors:** Chioma N. Tomlinson, Mara M. Hampson, Ankita Patil, Jacqueline Liu, Lisa DuBreuil, Janet W. Rich-Edwards

**Affiliations:** 1 Primary Care Center of Excellence, Department of Medicine, Brigham and Women’s Hospital, Boston, MA, United States; 2 Division of Women’s Health, Department of Medicine, Brigham and Women’s Hospital, Boston, MA, United States; 3 Harvard Medical School, Boston, MA, United States; 4 Massachusetts General Hospital, Boston, MA, United States; 5 Department of Medicine, Massachusetts General Hospital, Boston, MA, United States; 6 Department of Epidemiology, Harvard T.H. Chan School of Public Health, Boston, MA, United States

**Keywords:** weight bias and stigma, trauma informed approach, health at every size (HAES), multidiscipliary team, primary care

## Abstract

Weight stigma in healthcare contributes to poor patient outcomes, emotional harm, and avoidance of care. Healthcare systems are often perceived as hostile environments for many larger-bodied people who often report feeling judged, dismissed or denied appropriate treatment. Despite growing awareness, most medical educational programs and healthcare systems do not address weight bias directly. Persuading clinicians and staff to disrupt the traditional medical paradigm and instead adopt a size-inclusive perspective requires educational materials that push the envelope without pushing learners off a cliff. This paper describes the development of a weight-inclusive e-course designed to raise awareness of the impact of anti-fat bias in medicine. Grounded in the philosophical frameworks of Health at Every Size™ (HAES™) and Trauma-Informed Care (TIC), the course was co-created by a multidisciplinary team including clinicians, educators, activists, and individuals with lived experience. The collaborative process emphasized shared leadership, inclusive design, and emotional safety. We detail the course’s development over six months of weekly virtual meetings, including content creation, conflict resolution, accessibility planning, and evaluation design. The course includes three tracks tailored to clinicians, staff, and patients, and integrates practical tools for weight-neutral care. Lessons learned from this process offer a replicable model for inclusive curriculum design. Our aim is for learners to engage deeply with this work in order to fully reap the benefits for themselves and their patients. Institutions seeking to address weight stigma can use this framework to foster respectful, equitable care for people in all bodies.

## Introduction

Larger-bodied individuals face systemic bias in healthcare, often resulting in delayed diagnoses, emotional harm, and reduced trust. Traditional medical education rarely addresses weight stigma. This topic is also fraught with conflicting and seemingly incompatible points of view between entrenched clinical beliefs and an emerging movement of larger-bodied patients and fat activists who have found traditional healthcare systems unsympathetic, frustrating, and often harmful (1–3, 6, 7). This project aims to create a course that promotes a novel approach to respectful, evidence-based, weight-neutral care using Health at Every Size (HAES™) and Trauma-Informed Care (TIC) frameworks. Our team included professionals in clinical medicine, epidemiology, public health and social work. Our experience ranged from trainees to established clinicians and varied in body size, sexual orientation, and race. Collaboration across fields can be risky especially when personal and professional stakes are involved. This paper describes the process of team building and course creation by this diverse team. Our process centered lived experiences, challenged dominant narratives, and modeled inclusive design (10). We outline the barriers we’ve faced as well as solutions that made collaboration not only possible, but a source of personal and professional growth.

## The project

### Theoretical approach

This course was created for healthcare providers, trainees, staff, and patients seeking practical tools to recognize and reduce weight stigma. Our work is guided by HAES™ and TIC principles, to emphasize safety, dignity, and patient choice.

**Figure 1:**
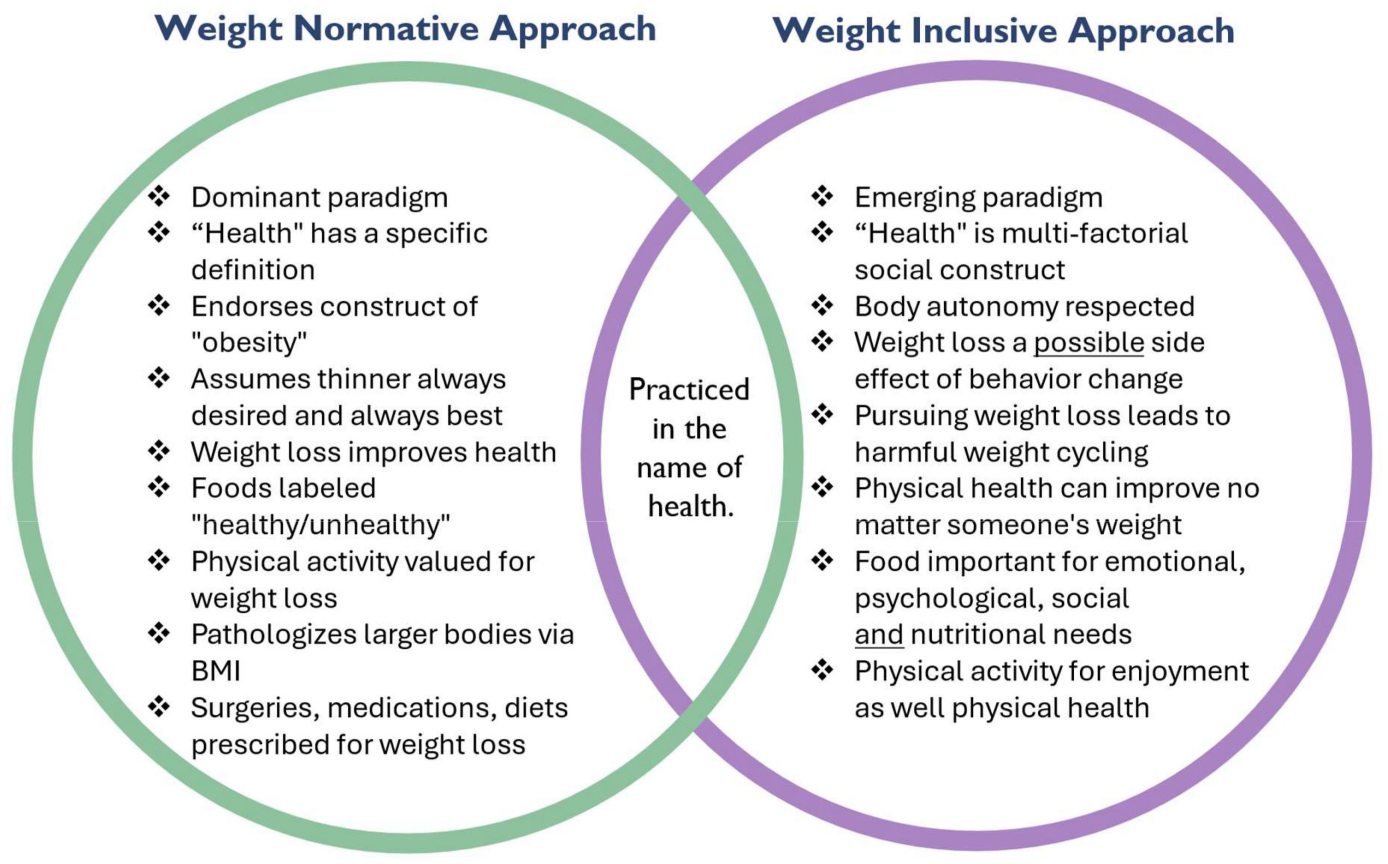
Excerpted script for this slide in the e-course: The “Weight Normative” perspective is the currently dominant view of weight: it proposes that people above specific BMI thresholds must lose weight to avoid various health complications. Therefore, a patient’s weight can become the primary health issue in a provider’s mind, regardless of their patient’s goals or reason for visiting. The fat body is seen as evidence of a problem, and obesity is perceived as an epidemic that must be solved. The Weight Inclusive Approach is a direct response to the weight normative perspective. Prioritizing the lived experience of individuals and celebrating body diversity, it questions BMI as a tool for indicating health for any given individual. Weight Inclusive advocates posit that people can be healthy at a wide range of weights and can improve their health without focusing on weight loss. Health At Every Size™ and other weight inclusive frameworks emphasize a people-centric and patient-led approach. Both the Weight Normative and Weight Inclusive perspectives are practiced in the name of health and share the goal of making people healthier and safer, while taking different approaches to that goal (4, 5, 9).

Health at Every Size (HAES™) principles challenge traditional weight-centric approaches to healthcare. Instead of pathologizing fatness and thus automatically attributing a higher weight to all adverse medical outcomes, HAES™ encourages the pursuit of health across a spectrum of body sizes and advocate that larger-bodied individuals deserve the same dignity and quality of care as anyone else (4). Our e-course contrasts the culturally dominant weight normative approach with weight inclusive approaches like Health at Every Size (**Figure 1**).

Trauma-Informed Care (TIC) principles recognize the widespread prevalence and impact of trauma and aim to minimize re-traumatization by creating safe, supportive environments. TIC emphasizes trust, empowerment, autonomy and collaboration while acknowledging historical/gender-specific trauma, cultural biases and systemic inequities. When applied in clinical settings, TIC often manifests as screening for trauma, awareness of triggers and giving patients choice (5, 9).

Together, HAES™ and TIC principles provide a framework to transform how larger-bodied patients experience healthcare by reducing the downstream effects of weight bias and stigma. When applied effectively, larger-bodied patients report feeling more respected, validated, safe and engaged in healthcare environments.

We made every effort to center the voices of those most affected by weight bias and challenge harmful norms in healthcare. This statement reflects our commitment to equity, not neutrality.

### Origins of the project

The team’s epidemiologist (JRE) is an academic in the Division of Women’s Health (DWH) at Brigham and Women’s Hospital (BWH) who had been working on studies linking child abuse to adult chronic disease; in her population data, the association was partly mediated by disordered eating and consequent ob*sity. She received pilot funding from the Lifecourse Research Network (LCRN) of the University of Los Angeles, California to translate research findings on early childhood adversity to a large-scale prevention project. These funds allowed her to convene a group of experts to advise on the design of a community intervention. The DWH administrative assistant (MH) helped prepare the workshop and proposed including the perspectives of fat activists. The workshop ultimately included activists, academics, clinicians and a representative from the Obesity Action Committee. Tensions arose almost immediately between an activist and a clinician over their perspectives on weight and its causes. Both later declined to advise the project. The input of the activists steered the project in a radically new direction, shifting the emphasis to the harm caused by often unbridled anti-fat bias in medicine. The workshop also revealed that anti-fat bias in medicine could be considered a source of trauma, and that there were few educational resources designed to address the issue.

Team formation: Identifying anti-fat bias as a source of trauma enabled us to adopt TIC practices that recognize the impact of trauma, reduce re-traumatization, and promote healing. We continued to reach out to the workshop participants for their advice. To learn more about TIC practices, we turned to experts within DWH (Drs. Annie Lewis-O’Connor and Eve Rittenberg) and to Harvard Medical School (Dr. Jennifer Potter) as early advisors to the project. The next step was to probe our network of clinical colleagues who could inform our clinical education. Chioma Tomlinson (CT) is a primary care provider well known for effective patient-centered care focused on improving outcomes for larger-bodied patients. CT was also familiar with applying HAES™ and TIC principles within her practice.

### Funding the e-course development

JRE, MH, and CT applied for the BWH Research Institute’s BRIght Futures Prize which is given to ‘answer provocative questions or solve grand problems.’ We successfully leveraged our personal and professional networks to win the prize in a competitive field. This allowed us to fund the assistance of Ankita Patil (AP) to translate the content into the online platform and along with MH conduct interviews with clinicians, community members and activists. Lisa DuBreuil (LD), a social worker and fat activist from Mass General Hospital (MGH) brought needed clarity and nuance to our understanding of HAES™ and continued to provide us with important references and contacts in the fat-positive community. Jackie Liu (JL) a second-year Harvard Medical School (HMS) student who had studied medicalized fatphobia as an undergraduate brought us insight into how medical students might receive our course.

### Other resources

Though we feared this topic and our approach might be too provocative for our own institutions, we have been well received at nearly every turn. We were fortunate to be well-supported by our institutions given this is typically not the case for work on anti-fat bias and stigma. Our professional credentials bought us credibility and afforded access to resources that would be inaccessible to activists without hospital and medical school affiliations. For instance, despite the change in project aims after the workshop, the LCRN leadership remained supportive. BWH Primary Care and DWH are home to many of the early pioneers in TIC who provided early counsel. The BWH Department of Primary Care wrote letters of support for our BRIght Future’s proposal and agreed to have us test our e-course at four clinics. When the BRIght Futures Prize reviewers cleared us to move forward in the competition, we were affirmed in our belief that our project was as valid and relevant as the typical basic science and clinical research proposals that won funding in the previous ten-year life of the prize. Division leadership at BWH provided time and flexibility for JRE and MH, as well as enthusiasm, expertise, and an opportunity to present our work to a national audience as part of the HMS Career Advancement and Leadership Skills for Women in Healthcare conference. Through JL’s advocacy at HMS, we were able to pilot the e-course with multiple cohorts of students. We took feedback from the pilot test with medical students to focus on the common core and to design the clinical track.

## E-course creation and evaluation process

**Figure 2:**
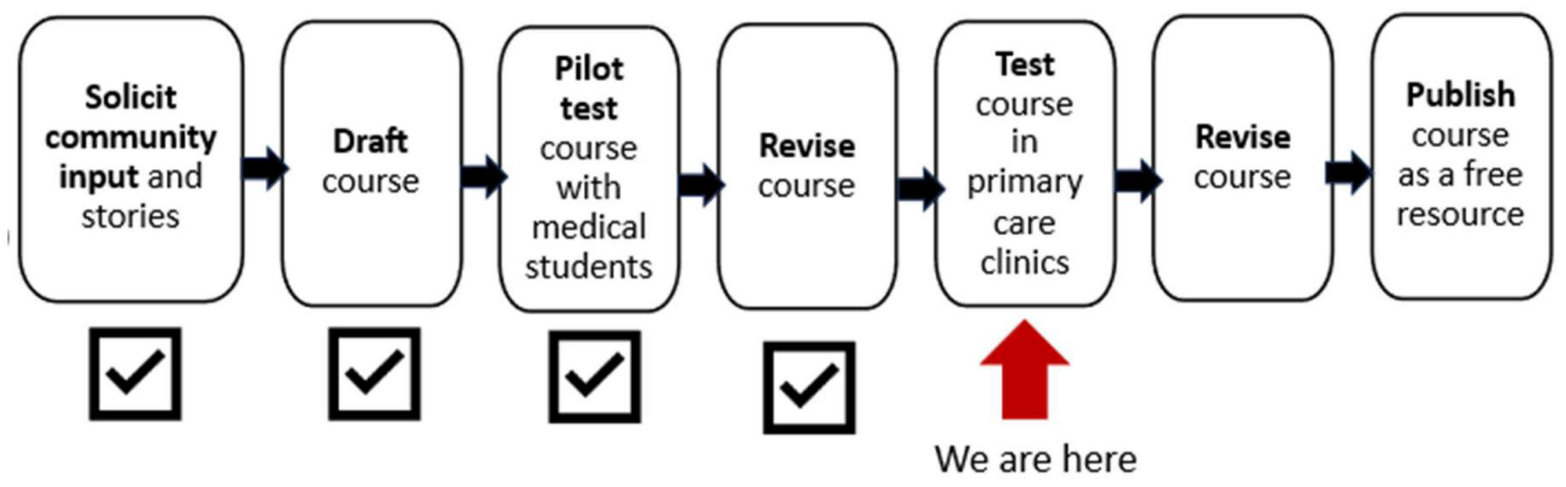
Timeline of e-course development, testing, and dissemination.

Originally conceived as a presentation, JRE and MH collaborated on a PowerPoint that could be given to various audiences of clinicians and community members. However, after seeing another e-course created by Dr. Brittany Charlton on LGBTQ+ experiences of stigma in the medical field, we realized that an e-course could be a more effective way to disseminate our message, because it would be more engaging and could be accessed online for asynchronous learning. With the BRIght Futures funding, we proposed to: 1) solicit the input and stories of patients in larger bodies, weight-inclusive clinicians and fat activists; 2) design an e-course incorporating their input with HAES™ and TIC principles; 3) test the e-course with clinicians; and 4) publish the course and its evaluation as an open resource (**Figure 2**).

At the outset, we realized that learning needs and styles may differ between clinicians and staff. We therefore proposed a course with two tracks, one for clinicians and the other for practice staff such as medical and administrative assistants. The need for a third track designed for patients arose during the process. At present, we have concluded the design of the clinician and practice staff tracks and have pilot-tested the clinician track with medical students (results in **Supplementary 1**). We are now preparing to test the e-course in two primary care clinics.

### Soliciting input

We invited participants in larger bodies via Instagram and word of mouth for semi-structured interviews regarding their experiences of living in a fat body and interacting with medical care. When given consent we recorded the Zoom interviews and extracted video or audio clips for the e-course. Participants chose whether to include their names along with their video (one declined) and if they wished to replace their image with an avatar to allow anonymity (none chose this option). We offered honoraria to community members regardless of their level of participation.

### Technology and accessibility

**Figure 3:**
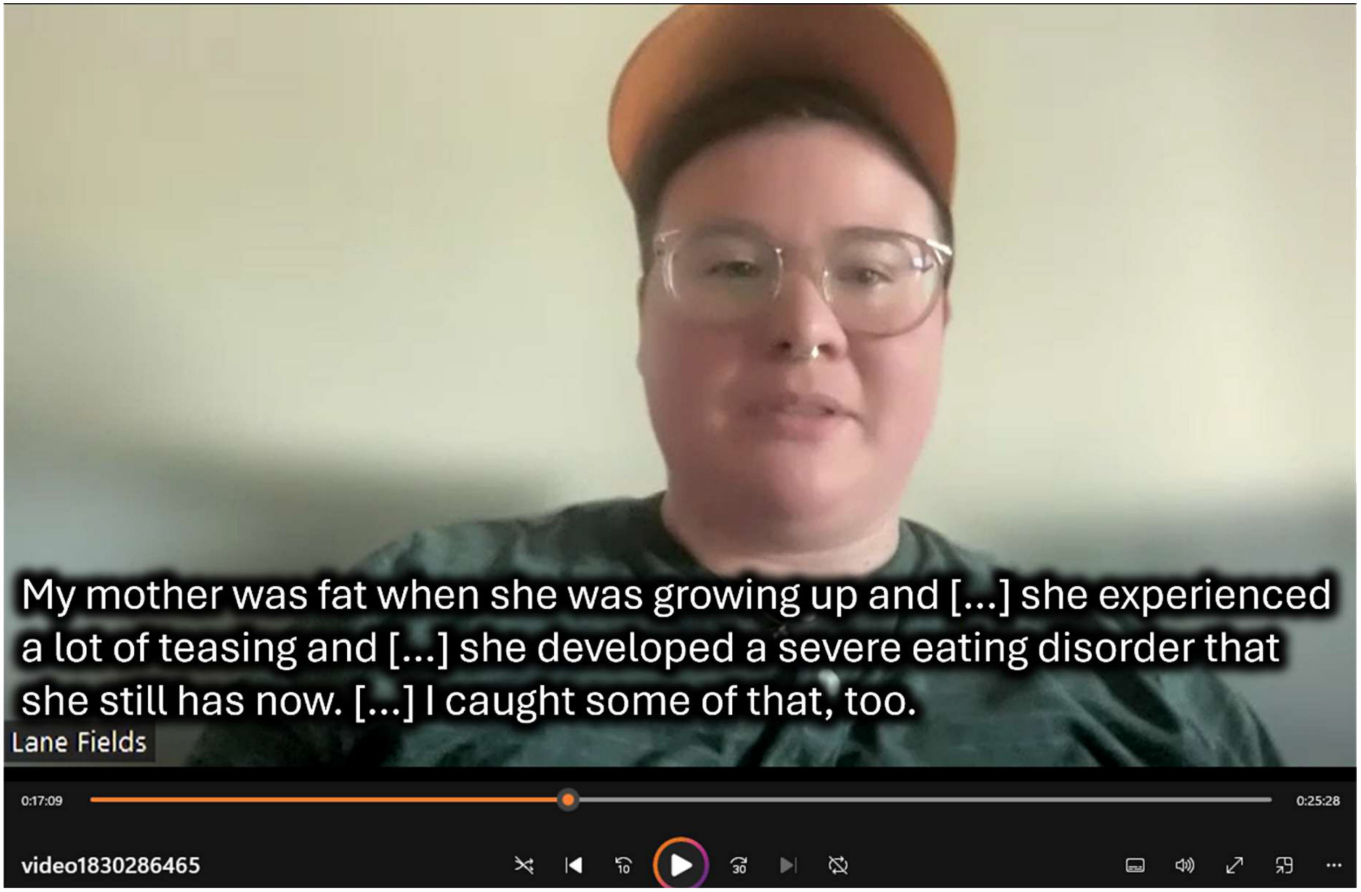
Screenshot from e-course of a community member interview (subtitles abridged for illustration).

We chose the Articulate platform for e-course development and deployment based on its flexibility, design options, and data tracking abilities. MH, AP and JL performed the iterative drafting and review process of the course content within the e-course platform, which featured closed captioning and screen-reader capability. The visual images and figures were deliberately intended to avoid stigmatizing imagery. **Figure 3** shows a screenshot of a community video that demonstrates the accessibility feature of subtitles.

### Meeting structure and workflow

**Figure 4:**
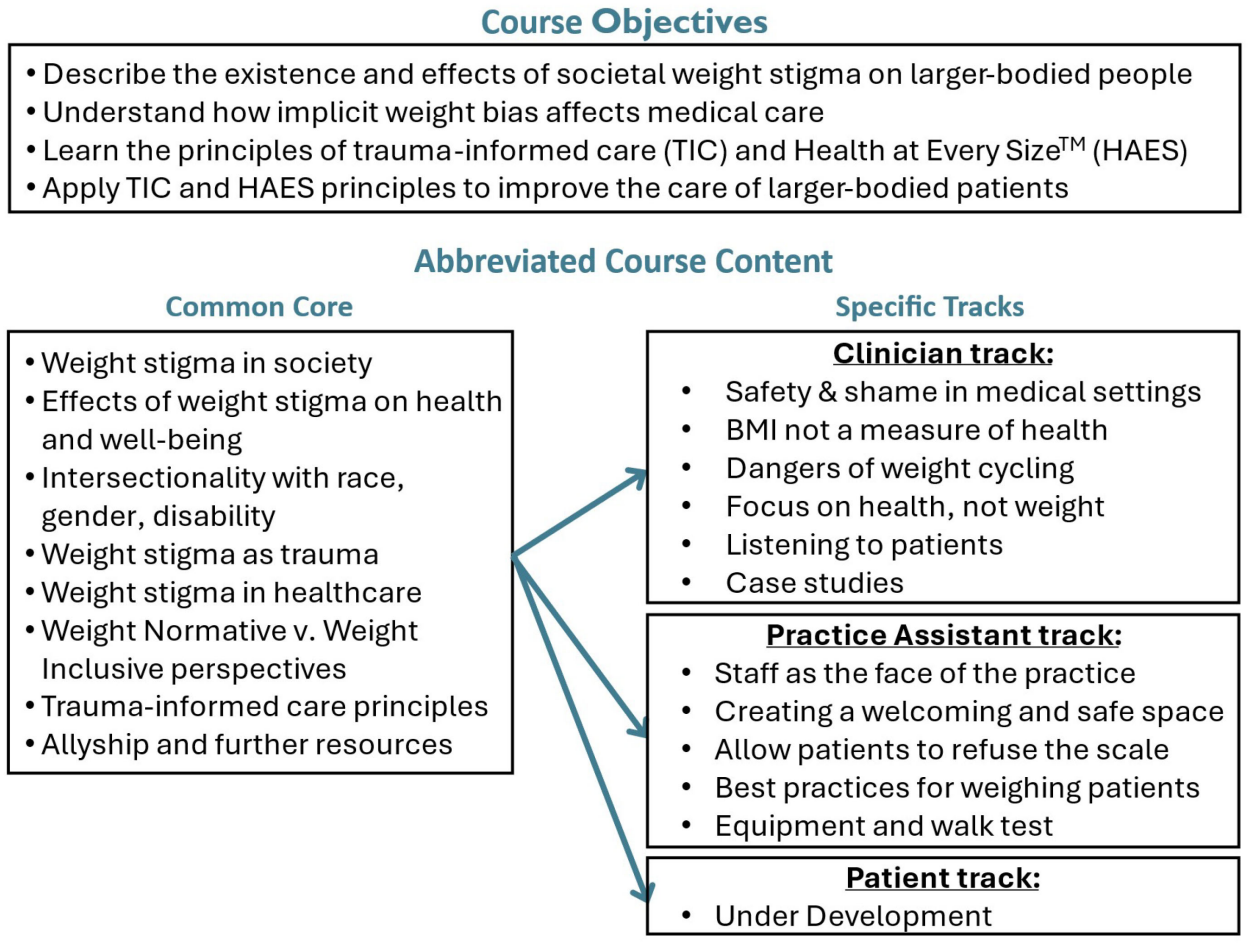
E-cource objectives and abbreviated content.

JRE, MH, CT and AP officially began construction of the e-course in June 2023. LD officially joined the team in September 2023 followed by JL in February 2024. Weekly 60–90 minute in-person and virtual meetings were held over 2 years. We began every meeting with check-ins to build trust, followed by updates, draft reviews, and problem-solving. Smaller groups worked between meetings on writing, design, and evaluation tasks, then shared progress with the full team. Written course content, interviews, sources and presentations were centrally located using DropBox™. **Figure 4** and **Supplementary 2** summarize the e-course content.

### Testing the e-course with clinicians

We have plans with several Mass General Brigham (MGB) primary care clinics to evaluate the course with clinicians and practice assistants this winter. We will seek resources to test the patient track once it is designed. **Supplementary 1** shows an example of the pre and post course surveys.

### Publishing the e-course and its evaluation

Assuming that the evaluation is generally positive and we can refine the e-course with minor improvements suggested by the evaluators, we will seek to publish the e-course on a peer-reviewed, open-source platform to make it widely available at no cost.

## Collaboration and conflict resolution

### Initial perspectives and positionality

Each team member brought perspectives foundational to course development. Here we capture each member’s initial perspectives and their evolution.

JRE (epidemiologist, she/her): I’m a white, cis-gendered, straight, woman scientist born in the Midwest in the 1960s to a life of thin privilege. I’ve studied the determinants of women’s health and chronic disease for nearly thirty years. Like my predecessors and contemporaries, I was trained that BMI is a strong determinant of health. I therefore believed a high BMI was something to prevent in childhood, since it was so hard to reverse in adulthood. I was startled early on by the candor with which a fat activist steered me from my focus on preventing ob*sity stating: ‘That’s not the real problem here.’ The experience opened my eyes to anti-fat bias and how plainly disrespectful, counter-productive and even traumatizing medical care can be for larger-bodied patients. As we approached the e-course, I was worried that my ignorance would hurt my colleagues. I was also wary that this project would ask me to deny my beliefs in the evidence regarding the associations of body size and health. I didn’t know if I could stand by my work and by my colleagues at the same time. While I still believe BMI impacts health, I now understand the futility of trying to create small people from large people and deeply appreciate the profound harms of medical and societal shaming of fat people.
MH (administrative assistant, she/her): I have been fat since I was a child. I have encountered anti-fat bias in nearly every venue possible, including church, family dinners, and most especially in the doctor’s office. While I’ve experienced prejudice my whole life, I never expected that it could risk my life until a doctor wrote off my acute abdominal pain as a signal that I “just” needed to lose weight. The next week I found myself in the ER with a ruptured ovarian cyst. That experience changed my perception of weight and what it meant about the person carrying it. Through friends I found an online blog that pointed out how society fails its fat members. I found a community of people that helped me reconceptualize what life could be like if, instead of hating my body and calling myself a failure, I could love my body for what it does for me. However, I was still so accustomed to being ignored or told that my opinion wasn’t valid. I was therefore astonished that an academic like JRE would listen to a non-academic like me, and that once I showed her the evidence of the harm anti-fat bias itself has on health, a partnership emerged which shifted the project’s emphasis to educate healthcare providers and staff about the downstream consequences of anti-fat bias. My voice was heard and that makes all the difference. I now consider myself a fat activist.
CT (primary care clinician, she/her): I am a first-generation African American. My cultural upbringing allows me to see beyond the typical western ideals of beauty and wellness. In 2013, two years into my career, the American Medical Association deemed “ob*sity” as a chronic disease. Over the ensuing years there was a palpable shift toward tackling this “epidemic” head on. By 2017 I began to develop a foundation in lifestyle medicine. The following year I started to explore the emerging field of ob*sity medicine. The dominant theme was that weight loss was a goal anyone could achieve at any time. This translated into a high degree of shame many patients in larger bodies would share with me about their struggle to lose and maintain weight loss. These personal stories fundamentally changed my approach to patient-centered care. As nutrient- stimulating hormone-based therapies for weight management have gained popularity and become ubiquitous within primary care, there has been an unfortunate sharp increase in anti-fat bias in patients and providers. When I was approached by JRE and MH to join their team I was eager to offer my clinical expertise and valuable insights on how to improve the clinical experience for both patients and providers. What challenged me early on was realizing that there was a whole community of activists that are strongly against medical treatment of any kind for weight management. I was initially wary of offering my options and anecdotes for fear I would be viewed as “one of them.” I now have a better understanding of how harmful experiences, often stemming from childhood, can be considered an independent risk factor for poor health outcomes in larger patients. Addressing anti-fat bias and stigma with weight neutral approaches is a matter of health equity and social justice.
AP (research assistant, she/her): Having worked closely with incarcerated individuals, I have seen how systemic oppression, stigma, and dehumanizing environments create lasting harm, making compassion and respect critical cornerstones of care. This work made me attentive to the ways social marginalization shapes health, but I had not fully considered how these dynamics extend to people in larger bodies. Joining this project led me to recognize the ways in which weight stigma parallels the very forms of marginalization I have witnessed in correctional settings – both rooted in societal bias, both perpetuating trauma, and both requiring a deliberate commitment to dignity in care. In confronting my own assumptions, I realized that weight stigma operates as its own pervasive and insidious form of oppression that demands its own framework of care.
LD (clinical social worker, fat activist, she/her): I’m a white, cis-gendered straight woman born in Boston in the 1960s to a working-class Irish-Catholic family. I’ve been in recovery from an eating disorder for 25 years and I am “super fat” – a term coined in 2008, essentially means most stores do not carry clothing in my size, and finding seating that works for me in restaurants, theatres and other public venues is often difficult. I work with people with substance use disorders and eating disorders and educate various groups of people about the impact of weight stigma on people’s health, especially in medical settings. At MGB I am part of the Size Diversity Group, a multi-disciplinary team that provides education and consultation to colleagues and patients about weight-inclusive medical care.

I first heard about the e-course project in May 2023. I remember being both very excited to hear about this project but also anxious as many similar projects don’t include any actual fat people or consider the importance of the language used to describe fat people. It was heartening to see that MH was highly involved, and I did not see (to my recollection) any use of the term ob*sity to disparage fat people. Encouraged, I reached out and very quickly got a warm email back with plans to meet virtually. When I viewed the draft material, it was clear the team did not have a good grip on the HAES™ approach. This is not unusual, in my experience it’s often difficult for people to get their heads around what to do to help fat people improve their health other than losing weight. There are also lots of misconceptions about HAES™ such as: a belief that weight has no impact on health or that food and exercise changes cannot improve health.

Given the clear commitment this team had to this project I drafted an email explaining my concerns and offering to assist them in understanding the HAES™ model. Once again, I got a quick and friendly reply taking me up on my offer. What was initially going to be a couple of consultation meetings turned into weekly zooms for the past 16 months. When doing this kind of activism, I always have to balance how anxiety-provoking engaging can be with the positive outcomes that can come from this work. While our early meetings often left me uneasy and fatigued, I was able to develop the necessary coping skills to make sure the HAES™ model was accurately represented and that the e-course was informed by the actual experts on the best medical care for fat people – which are fat people. Eventually I learned to trust and moved from someone who was trying to act as a type of guardrail to a real member of the team creating a first of its kind e-course. I gained a deeper understanding and appreciation for the challenges of providing weight-inclusive, trauma-informed care in clinical settings especially when patients want to lose weight.

JL (medical student, she/they): I have been a thin person my whole life. I used to pride myself on my below average BMI. During that time, I restricted my food intake, over-exercised, and obsessed over the way my body looked. I was introduced to the notion of fatphobia as an undergraduate in my first gender studies class. That class allowed me to question the presumptions I had about larger bodies being unhealthy and undesirable. It made me confront the futile goal I had been pursuing of achieving the “ideal” and began the process of healing my relationship with my body. As I learned more about the research on medicalized weight stigma and bias, I knew that I wanted to pursue a career in medicine and dedicate my practice towards ending weight bias and stigma. I decided to write my senior thesis on this topic. There, I proposed a medical environment devoid of subpar care for larger-bodied people, where each patient is empowered to lead a life that they find satisfying and whole.

In my first year of medical school, I was confronted with the standard messaging that weight is a risk factor for several medical pathologies. What was lacking however was pathophysiological mechanism or epidemiological evidence; instead, there is often an automatic presumption that fatness equates to bad health. I found it difficult to continuously fight against the traditional paradigm. It became even muddier when I would talk to patients who lamented their body size or arbitrarily connected all their medical conditions to their weight. Who am I to try and correct them, especially when we live in such a fatphobic world? I joined the project to provide my perspective as a future clinician. I wholeheartedly agreed there needed to be a change to the fundamental way healthcare providers are trained to think about patients in larger bodies. As the team engaged in sticky discussions on how fat patients should be counseled, I was able to appreciate the nuance that surfaces when practitioners from different fields weigh in on this topic. After working with this team, I’ve learned what it takes to change minds and practice, as well as how incremental change is still just as meaningful as sweeping reform.

### Trust-building, consensus and conflict

A significant challenge was to create space for opposing viewpoints, particularly regarding the tone and content of the course. Anti-fat bias and stigma are commonly an emotionally charged space. HAES™ activists are often labeled as “angry,” a harmful stereotype also shared by women of color. Finding common ground could not be assumed. We slowly built trust through several factors. On the one hand, while we acknowledged the need to deliver updated content to individuals already familiar with concepts of anti-fat bias, intersectionality, and hierarchies in medicine, we also needed to speak to healthcare professionals and patients firmly rooted in the prevailing zeitgeist of the ‘ob*sity epidemic.’ Our team often had divergent views of how to meet the traditional viewpoints of more conservative learners while presenting the evidence to convince staff and providers to abandon the BMI as a sole predictor of health, to refrain from attributing every complaint to a patient’s size, and to appreciate the expertise that every person has of their own body. MH and LD in particular took risks by sharing personal examples from their lives, since larger-bodied people are often not believed when they recount painful experiences. We each ventured opinions from our own disciplines while stretching to meet the others where they stood in their core beliefs. By listening carefully, we learned to express our feelings and speak our beliefs without shutting down the conversation and impede compromise. As the team successfully navigated these differences, we quickly established a shared understanding that anti-fat bias in medicine is a social determinant of health and agreed to focus on reducing its pervasiveness and harm. When we disagreed, we kept returning our focus to the touchstone of reducing bias, not teaching medicine. Our purpose was not to convince clinicians and staff that increased adiposity is harmless, but to teach them that an unthinking and biased approach is harmful and counterproductive. Through structured dialogue, silent reflections and shared values, we also leveraged the team’s diversity of race, body size and profession to inform course content. We fully acknowledge, however, the limitation that we all live in female bodies.

Conflicts were expected and handled with care. When tensions ran high, team members reached out individually to support each other and clarify concerns. Our shared goal—reducing anti-fat bias in healthcare—helped us stay focused and move forward together. We each respected the particular expertise of the other team members. Patient perspectives were informed mostly by MH and LD. They also ensured course content aligned with HAES™ and TIC principles. CT provided insight into clinical recommendations and strategies while JRE ensured adherence to research guidelines and ethics as well as devised pre/post evaluation assessments.

## Summary of barriers to replicability

Institutional Support and Resources: The content challenged dominant medical paradigms and risked institutional pushback. The project benefited from unusually strong institutional backing. Others may struggle to get similar backing, especially without medical or academic connections and in more traditional environments.

Team Composition and Diversity: The collaborative process relied heavily on a multidisciplinary team with diverse lived experiences, including clinicians, educators, activists, and patients which may be challenging to replicate in other settings.

Trust-Building and Conflict Resolution: Building trust among team members required a strong commitment to structured dialogue and sometimes difficult conversations and significant time for consensus-building which may be difficult to achieve in other settings.

Emotional Labor and Risk-Taking: Team members with lived experience of weight stigma took personal and professional risks by sharing their stories and perspectives. Sustaining this level of vulnerability can be a barrier to others attempting similar work.

Access to Funding and Technology: The ability to secure competitive grants and access user-friendly technology platforms (like Articulate) facilitated course creation and dissemination.

## Discussion

We are aware that there is still a long way to go toward treating all people with dignity and respect in all settings, always. Many academic medical centers remain steeped in a strong culture of treating “ob*sity” as a disease requiring aggressive action (1). Our goal is not just to change the conversation; we want to see an entire paradigm shift. Our project exemplifies hope for progress in understanding and addressing the unique barriers larger-bodied patients face when accessing medical care in this charged landscape (2, 6, 7). We acknowledge we were exceptionally lucky to find each other; this type of coalition building is not only possible but necessary to advance health equity. This course is more than a training tool: it’s a step toward changing how healthcare treats larger-bodied people. By combining lived experience with clinical insight and grounding our learnings in HAES™ and TIC, we created a replicable model for equity-driven curriculum design that is practical, inclusive, and adaptable (8). We know this is just the beginning. Our hope is that this course sparks deliberate reflection, shifts practices, and opens doors to more respectful and compassionate care that fosters resilience and healing—for everyone, in every body. Creating this course was both challenging and rewarding. Team members brought different experiences, ideas, and emotions to the table. Some had lived through medical weight stigma; others were learning about it for the first time. These differences sometimes led to tension, but they also made the course stronger. What kept us moving forward was a shared goal: to make healthcare safer and more respectful for people in larger bodies. We learned to listen deeply, speak honestly, and stay open to change. The process wasn’t perfect, but it was real—and it reflected the kind of care we hope to inspire.

## Data Availability

The original contributions presented in the study are included in the article/**Supplementary Material**. Further inquiries can be directed to the corresponding author.
