# Supplementary material for: Chutes and ladders: collaborating across disciplines to improve mental and physical healthcare for larger-bodied people
Source: Front Psychiatry. 2025 Nov 18;16:1589858. doi: 10.3389/fpsyt.2025.1589858 (PMC12746646; doi:10.3389/fpsyt.2025.1589858)
Supplement: Supplementary file 1 [file DataSheet1.pdf]

Supplement 1: Pilot Test of Common Core Module with Medical Students

Purpose: To pilot test a draft version of the common core module of the e-course, Trauma-Informed Care for Patients with Larger Bodies, with second year medical students. We planned to use their feedback to revise the core module and prepare the applied, skill-oriented tracks for clinicians, practice assistants and patients.

Methods: We emailed second-year Harvard Medical School students to enroll in the course and complete pre- and post-course surveys. Students had completed their preclinical curriculum and were preparing to enter into their year of clerkship rotations. To promote candor, we made the REDCap survey anonymous by instructing participants to create a unique ID number that could be recreated for the post-survey (see surveys in Figure S.1).

Three questions repeated in both the pre- and post-course surveys asked participants about their familiarity with the concept of anti-fat bias, the frequency of discrimination against larger-bodied people, and how much of a problem anti-fat bias presents in healthcare. The response option for these items was a slider scale (0-100) anchored at each end by descriptors such as 'Not at all' and 'Extremely.'

Three questions in the post-course survey queried how much information was learned, whether participants would recommend the course to other medical students and clinical colleagues, and how much they thought the course would affect their approach to clinical practice. Responses were from four- to six category Likert scale (see below).

Open ended text boxes were used to ask how participants might change their clinical practice and to solicit strengths and limitations of the e-course.

As responses were skewed, we examined median responses to survey items and median change in repeated items. Pre- and post-course responses were compared statistically with Wilcoxon signed rank tests.

Results: Of 127 students invited, 61 took the pre-test survey and 51 the post-test survey; we were able to link pre-test and post-test results of 43 students (several created more than one unique ID number which precluded examining change across surveys).

Figure S.2 displays the results of the three questions repeated before and after the course regarding familiarity with anti-fat bias, the frequency of discrimination against larger-bodied/fat people, and the degree to which anti-fat bias is a problem in healthcare. All three questions had high median pre-course scores, ranging from 72 to 91. Despite the high baseline scores, we observed increased scores in the post-course survey responses. Within-person change was greatest for familiarity with anti-fat bias, with a median 10-point

increase (p<0.0001). The median change was also positive for anti-fat bias in healthcare, increasing by 3 points (p<0.0001). Although the median change in the response to the question ‘how common is discrimination against larger-bodied/fat people’ was 0, it was nevertheless statistically significant, (p<0.0001), reflecting the increase in scores among people who had not responded at the top of the scale before the survey. Accordingly, when we reanalyzed this item excluding the 18 respondents who had scored 95 or higher on the pre-course survey, the median change was +9 points.

Figure S.3 displays the results of the post-survey questions. The median for all three items was 3, corresponding to “a moderate amount” in response to the question about how much was learned, “probably yes” in response to the question about recommending the course, and “a moderate amount” to how much students anticipated the course would affect their clinical practice.

The comments of students in response to the open-ended questions are displayed in Tables S.1 through S.3. Among those who indicated that they would change their practice, students said that they would be more conscious of the language they used and their internal biases. They appreciated the TIC approach to combat inequities in care (Table S.1). They found the patient testimonies engaging and informative (Table S.2). With respect to improving the course, participants requested more specific practical translations of the Common Core material to clinical practice, with one acknowledging this was the topic of the next module (Table S.3)

<u>Limitations</u>: Just under half (48%) of the second-year medical students elected to take the course, which was voluntary and offered without incentive. The ID system we employed to keep the survey anonymous may have been inadequately explained, resulting in several pre-course and post-course survey responses that could not be matched. Several of the survey items demonstrated ‘ceiling effect’ challenges, in that the pre-course scores were so high that there was little room to detect improvement. Before any course, students may accurately rate their own knowledge as high or may overrate it due to social desirability bias.

<u>Discussion</u>: In this pilot test of a new e-course on trauma-informed care for patients with larger bodies, we received mixed feedback. On the one hand, student awareness of anti-fat bias in society and in the healthcare system improved considerably, despite the insensitivity due to the ‘ceiling effect’ of some of our measures. On the other hand, we received only modest enthusiasm to recommend the course to colleagues. Interpreting these scores in light of the comments, we took away that these students had been conversant with issues of anti-fat bias prior to the course and were eager to move from the

‘consciousness raising’ material to practical methods to mitigate anti-fat bias in their clinical practices.

We used this feedback to revise the Common Core and as a guide in creating new material for the applied tracks for clinicians, practice assistants, and patients. Although we maintained much of the material in the Common Core, aware that this sample of medical students were likely more aware of anti-fat bias than practicing clinicians in the community might be, we did move some of the material to course appendices. We maintained patient testimony and added clinical vignettes with specific scenarios and communication techniques. Finally, the feedback allowed us to focus the remainder of the course on practical approaches for clinicians and practice assistants.

Figure S.1 The pre-course and post-course surveys for the pilot study

Pre-course Survey

Please complete the survey below. We will use your answers to evaluate the ecourse (not you!)

Thank you!

---

1) Please create your unique ID number: using all caps, type the first 2 letters of your mother's first name, the month of your birth, and the last 2 digits of your social security number: For example, if your mother's name is Lila, you were born in September, and your SSN is ***-**-**81, your ID would be: LI0981. We will use this to link your pre-course survey with your post-course survey and preserve your anonymity.

   ____

2) How familiar are you with the concept of anti-fat bias?

   Not at all familiar — Very familiar

   (Place a mark on the scale above)

3) How common is discrimination against larger-boded/fat people?

   Not at all — Extremely

   (Place a mark on the scale above)

4) How much is anti-fat bias a problem in healthcare?

   Not at all — Very much

   (Place a mark on the scale above)

Post-course Survey

Thanks for taking the ecourse. We ask that you complete this short survey to give us feedback we can use to improve it for future students and for use in clinical settings

Thank you!

1) Please create your unique ID number: using all caps, type the first 2 letters of your mother's first name, the month of your birth, and the last 2 digits of your social security number: For example, if your mother's name is Lila, you were born in September, and your SSN is ***-**-**81, your ID would be: LI0981. We will use this to link your pre-course survey with your post-course survey and preserve your anonymity.

____

2) How familiar are you with the concept of anti-fat bias?

Not at all familiar — Very familiar

(Place a mark on the scale above)

3) How common is discrimination against larger-bodied/fat people?

Not at all — Extremely

(Place a mark on the scale above)

4) How much is anti-fat bias a problem in healthcare?

Not at all — Very much

(Place a mark on the scale above)

5) How much information did you learn from this course?

- ◯ Nothing
- ◯ A little
- ◯ A moderate amount
- ◯ Quite a bit
- ◯ A lot
- ◯ A great deal

6) Would you recommend this course to other medical students and clinical colleagues?

- ◯ Definitely not
- ◯ Probably not
- ◯ Probably yes
- ◯ Definitely yes

7) How much do you think this course will affect your approach to clinical practice?

- ◯ Not at all
- ◯ A little
- ◯ A moderate amount
- ◯ Quite a bit
- ◯ A lot
- ◯ A great deal

8) Please comment on how this course might affect your approach to clinical practice:

____

We'll use your input to revise this course before we test it among primary care clinicians. We'd really value both your positive and negative feedback.

9) Please comment on the strengths of the course:

____

10) Please comment on how the course could be improved:

Figure S.2 Results of pre- and post-course survey of medical students for the pilot test of the e-course 'Trauma-Informed Care for Patients with Larger Bodies'

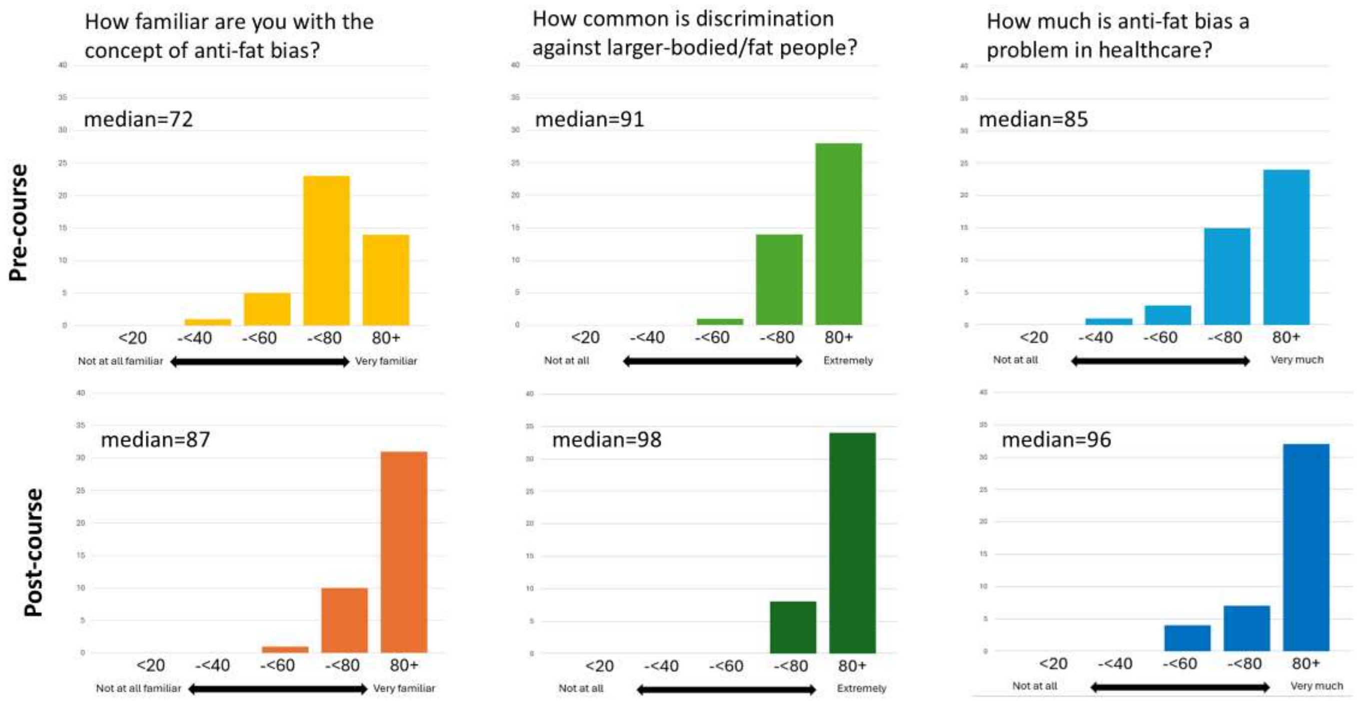

Figure S.3 Results of pre- and post-course survey of medical students for the pilot test of the e-course 'Trauma-Informed Care for Patients with Larger Bodies'

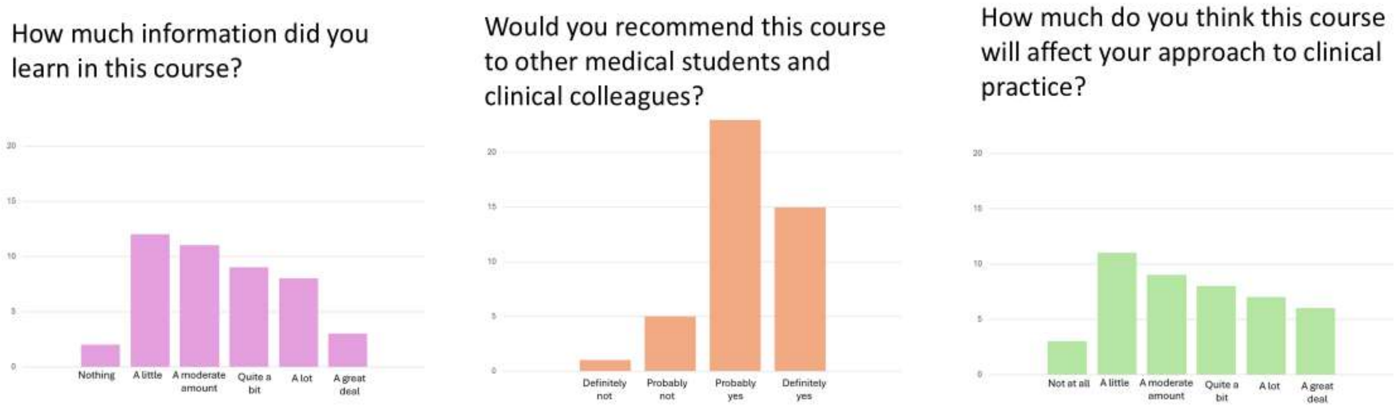

Table S.1 Open text responses to the question 'Please comment on how this course might affect your approach to clinical practice.'

I hope to be cognisant of the themes highlighted in the course, avoid retraumatizing patients and try to provide trauma informed care when working with people of large size.
i will be more aware of language I use and internal biases
More conscious of language used and being an upstander.
I'm really glad this is available! I will be able to cite these statistics to fellow clinicans
I will try to focus on using language that makes patients of different body sizes feel comfortable.
Thinking more consciously about screening for alternative differential diagnoses instead of just attributing it to weight, which is similar to how people with disabilities may be treated in the healthcare system
It's always good to be mindful of other peoples' experiences and struggles.
I think I was always well aware of fat bias and the importance of dispelling this for people of all shapes and sizes. But after this course I have realized just how prevalent and damaging anti-fat bias can be, and will be more on my guard about educating others in my workplace about this.
I'm trying my best to unlearn the weight stigma baked into medical school curriculum in order to advance equitable care for patients!
I learned about how to create a TIC environment for patients with larger bodies, which includes both clinician communication/behaviors and physical structures in the healthcare environment.
I want to create a safe space for any future patients I may have who identify as fat or overweight - that can include asking what language they prefer, asking what is important to them (and not discussing weight unless it is something they bring up or it is posing a significant risk to their health), using a trauma-informed approach. This module helped me understand what it's like to navigate life and the healthcare system as someone who is overweight, and I want to do my best to minimize harm and welcome patients of any and all weights, and treat everyone with respect.
I have seen a lot of emphasis on BMI even just in the epic platform, so I like the idea of using a health-at-any-size approach and working with patients to understand what they need.
It reminded me of the multiple ways in which fatphobia can appear within clinical settings, from gowns to equipment. It also reminded me about the importance of engaging patients around what matters most to them.
I will think more about how stigma affects all regardless of BMI

Table S.2 Open text responses to the question 'Please comment on the strengths of the course.'

Very direct and to the point! Easy to follow and provides accurate facts paired with great first hand stories from patients and physicians
Patient anecdotes are always very powerful and I appreciated that here
Good introduction and easy to understand
great intro course
It was interesting to read about representations of larger bodies in fiction.
Information presented in a variety of forms. Engaging and interactive.
I like that there were so many quotes from actual fat people and patients explaining concepts in their own words. Another strength was the specific examples of thin privilege in everyday life (public transit, restaurants, etc.) and weight discrimination in healthcare settings.
I think the course was more engaging because it was divided into smaller sections with shorter video clips and limited text presented at once.
Really enjoyed the videos of patients and physicians discussing their experiences. Also liked how there were written transcripts below the recorded audio clips.
It clearly laid out the issues around fatphobia and bias, and how they may affect medical care.
I loved the exposure to language and patient perspectives
I loved the inclusion of patient voices and activists and the history, involving PoC and queerness

Table S.3 Open text responses to the question 'Please comment on how the course could be improved.'

More alternatives / examples of what biased vs non biased behavior looks like so students can have more tangible takeaways
more specific examples of scenarios you might face and how to respond as a healthcare provider
I think some of the sections could be shortened!
Maybe quizzes in the middle to reinforce information retention.
I would love to see examples or get advice on how to disrupt bias and stigma among clinical colleagues. I've been in so many situations where I suspect that a doctor is responding from a place of anti-fatness and I've tried to gently question them only to be shut down by defensiveness. I'm wondering how much of that comes from messaging that clinicians have internalized, viewing themselves as morally good or disciplined because of their own health and thin privilege. Anyway much to think about, great overall module, maybe one of the more useful things I've learned at HMS so far.
It would be great to have more infographics included in the presentation to accompany the text.
I think it could have considered how weight and health have been "historically" related, and what ought to specifically change. Weight is still presented as a risk factor for many conditions. When does it mater? When should that be discussed. Also, it would be important to address recent pharmaceutical development such as GLP1 inhibitors.
I think there needs to be some sort of interactive component here, this is all very helpful information, but I wonder how to apply it when the norms of medicine are not there yet, when our preceptors don't agree with us, etcetera. And also when we don't know what it means to practically apply what was discussed in these modules. Awareness building is not enough.
I would love to see more clinical connections, maybe that is more in part 2!
