# Supplementary material for: Chutes and ladders: collaborating across disciplines to improve mental and physical healthcare for larger-bodied people
Source: Front Psychiatry. 2025 Nov 18;16:1589858. doi: 10.3389/fpsyt.2025.1589858 (PMC12746646; doi:10.3389/fpsyt.2025.1589858)
Supplement: Supplementary file 2 [file DataSheet2.docx]

**Supplement 2: Illustrative content from the e-course**

Section A. Content from the common core for clinicians, practice assistants and patients


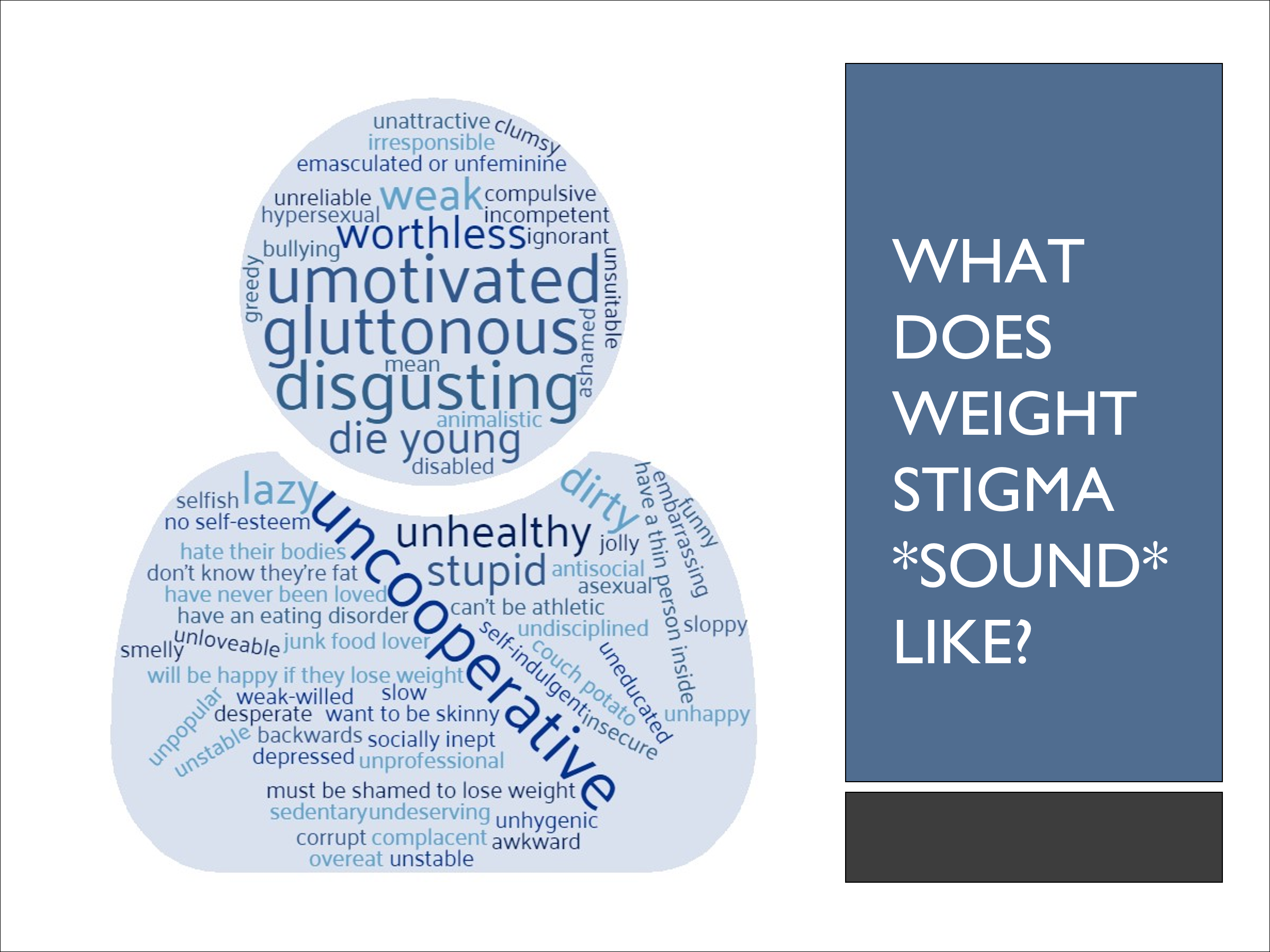


Figure S.1 Example of course content illuminating societal weight stigma.

Course script: There are many stereotypes about fat people; some are contradictory, but most are based on the idea that a person can only be fat if they are failing in some way: they are too stupid to exercise, too greedy to eat healthily, or too self-absorbed to realize what they look like and how people think about them. They have a thin person inside them just waiting to be free. They would be so pretty if they just lost some weight. They wouldn’t hate themselves so much if they could just summon the self-respect to live in a healthy way. When people look at a fat person, they often assume that they know many things about that person: that they eat too much, they move their bodies as little as possible, and they will die young. And often, out of a misplaced sense of concern or, in the worst case, a perverse delight in a type of socially sanctioned bullying, people around a fat person will try to use those stereotypes to convince them to change. No amount of evidence to the contrary will convince them that a fat person can be happy, successful or taking good care of their health.


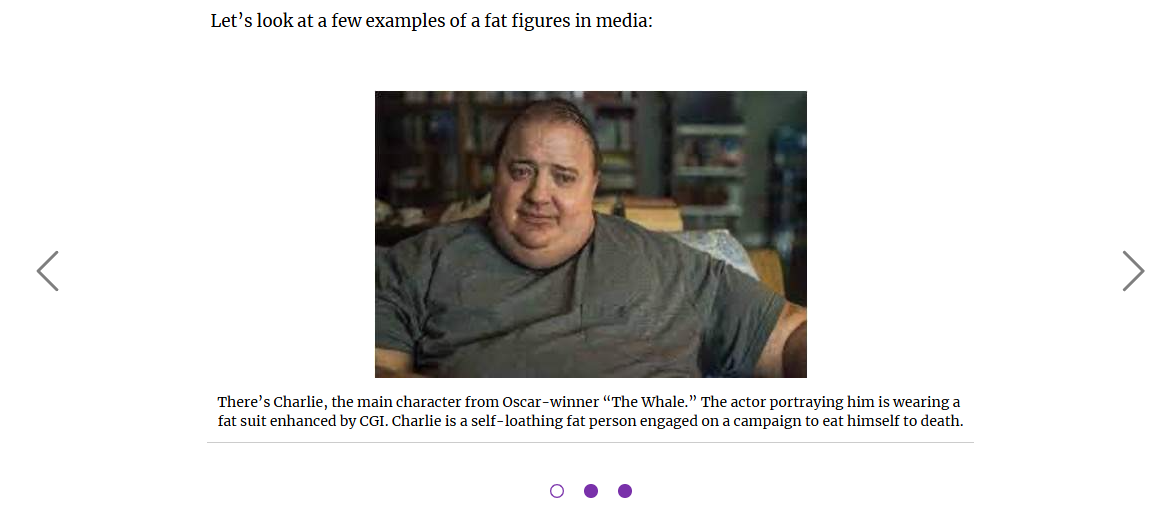
Figure S.2 Example of course content illuminating societal weight stigma in popular culture.


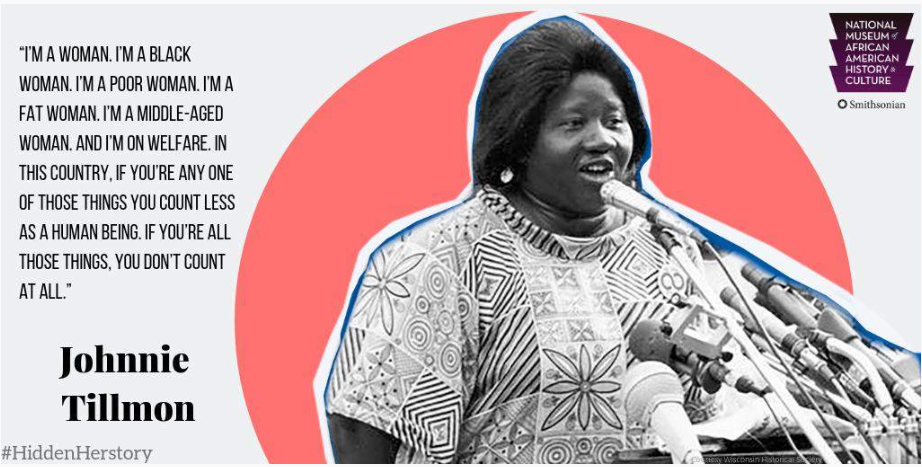


Figure S.3 Example of course content illuminating intersectionality of weight stigma and other forms of discrimination


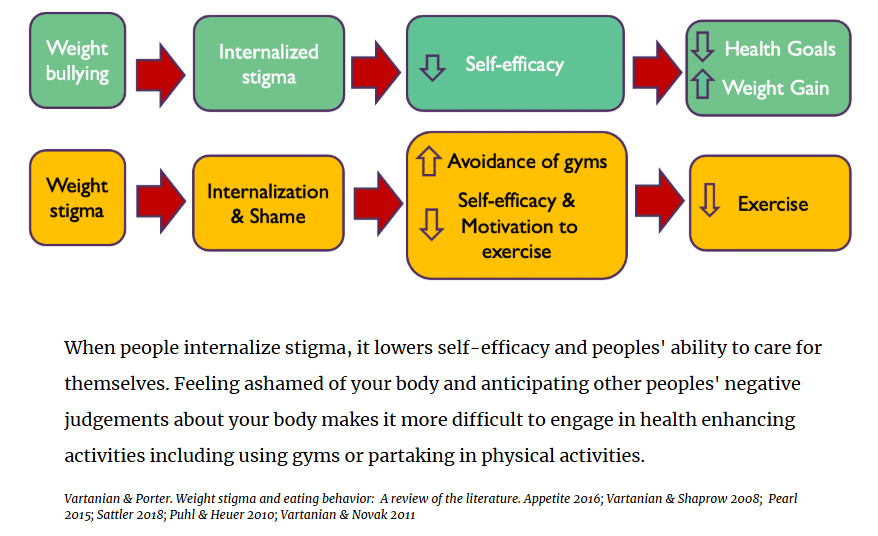


Figure S.4 Example of course content regarding internalized stigma


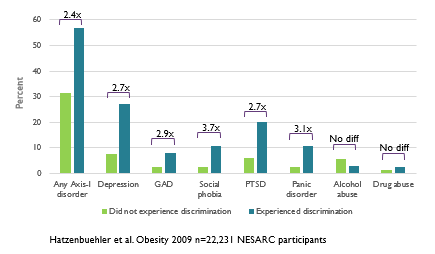


Figure S.5 Example of course content contrasting approaches to weight.

Course script: Like any form of discrimination, weight stigma affects mental health. This is clearly seen in a national study of over 22,000 participants. People who reported experiencing weight stigma (shown here in the darker bars) had between 2- and 4-times higher risk of depression, generalized anxiety disorder, social phobia, PTSD, and panic disorder than people who did not experience weight stigma. Notably, these differences persisted after accounting for BMI. In other words, for two people of the exact same BMI, the person who has experienced weight stigma is at a much higher risk of poor mental health.


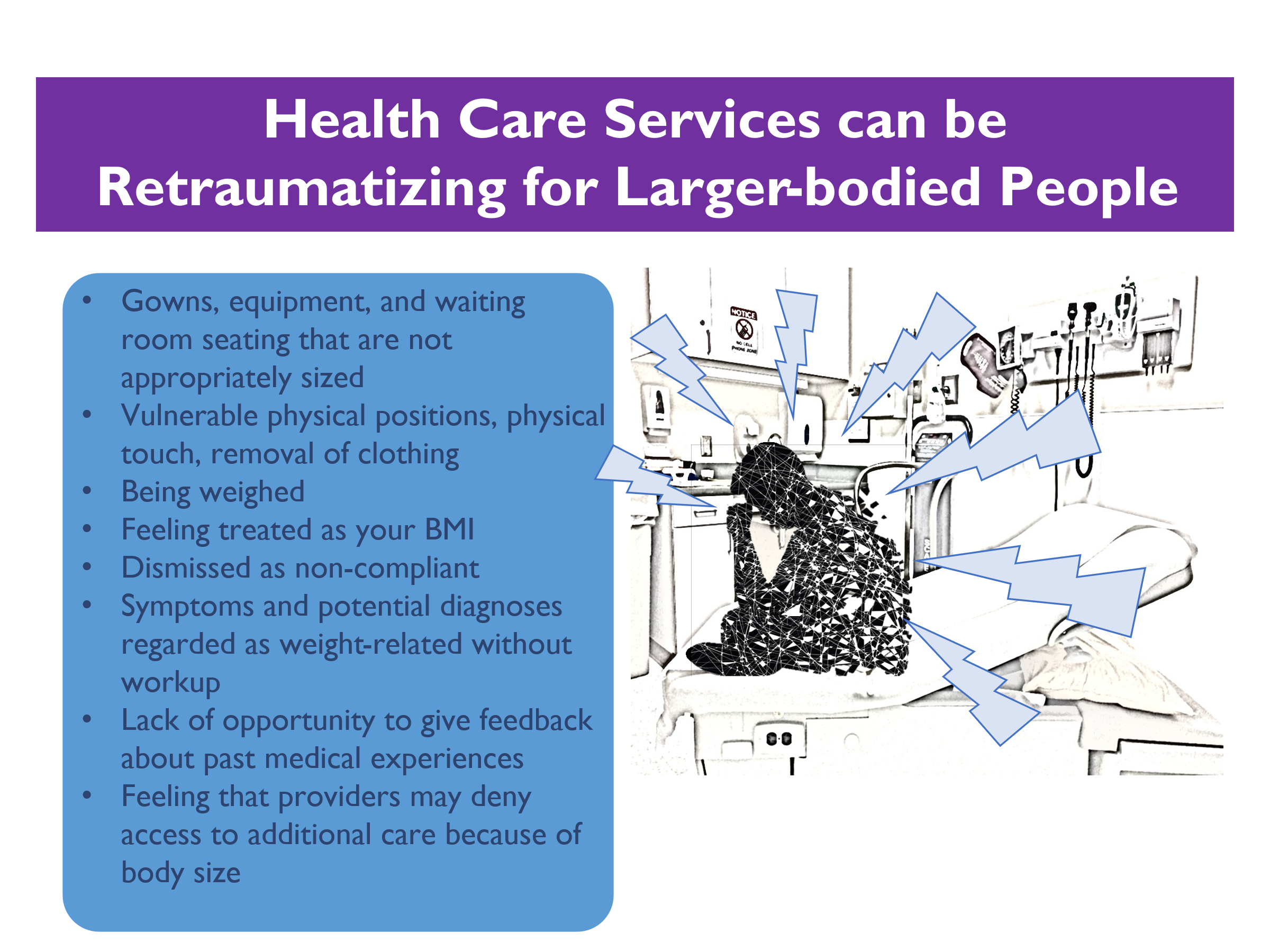


Figure S.6 Example of course content introducing weight bias in healthcare as a source of trauma

Course script: Remember, when a person of size enters your clinic, they bring with them all their experiences, sometimes a lifetime of experiences of weight bias, disrespect, and discrimination, including in medical settings. Those experiences are almost always an invisible presence in the room with the provider and the patient.


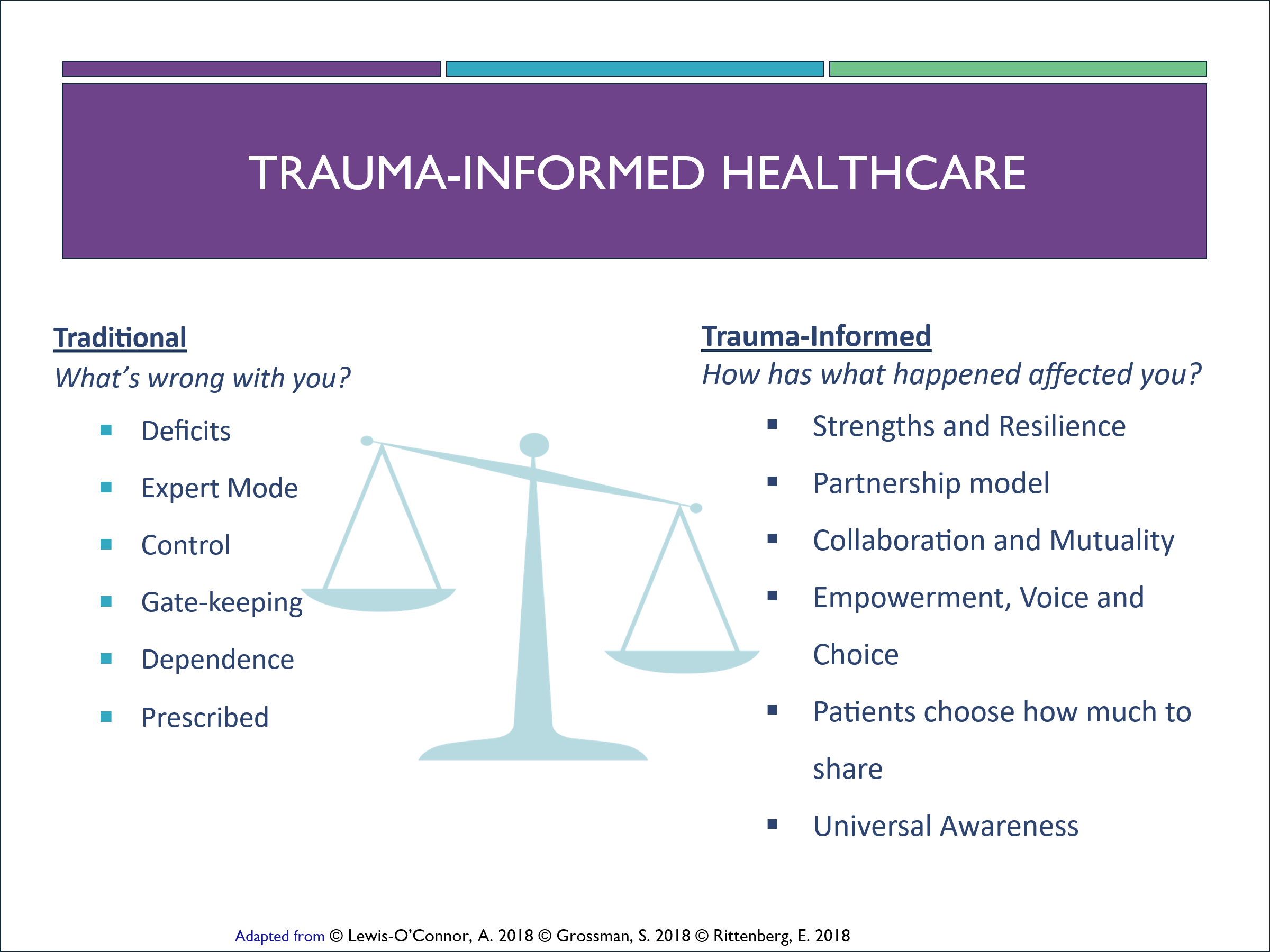


Figure S.7 Example of course content introducing trauma-informed healthcare

Course script: Trauma-informed healthcare requires a philosophical shift. The traditional paradigm asks of a patient, "what's wrong with you?" This framework emphasizes deficits and assumes the provider has power as the expert and as someone with control over the patient. Within this framework, the provider knows more about the patient's body than they do. The provider serves as a gatekeeper to health and further care, creating an innate dependence of the patient on the provider. There is a stark power differential and no process for acknowledging a patient's background or the trauma they may have experienced.

The shift to a trauma-informed perspective asks the patient, "how has what has happened affected you?" The patient's strengths and resilience are honored and emphasized. The patient and provider are seen to have a partnership in which they practice collaboration and mutuality. In a TIC framework, the patient has empowerment, voice and choice within the clinical setting. The patient guides their care and chooses how much of their trauma history they want to share. Additionally, this framework acknowledges the principle of Universal Awareness, in which the provider recognizes that almost every patient has a trauma history of one kind or another.


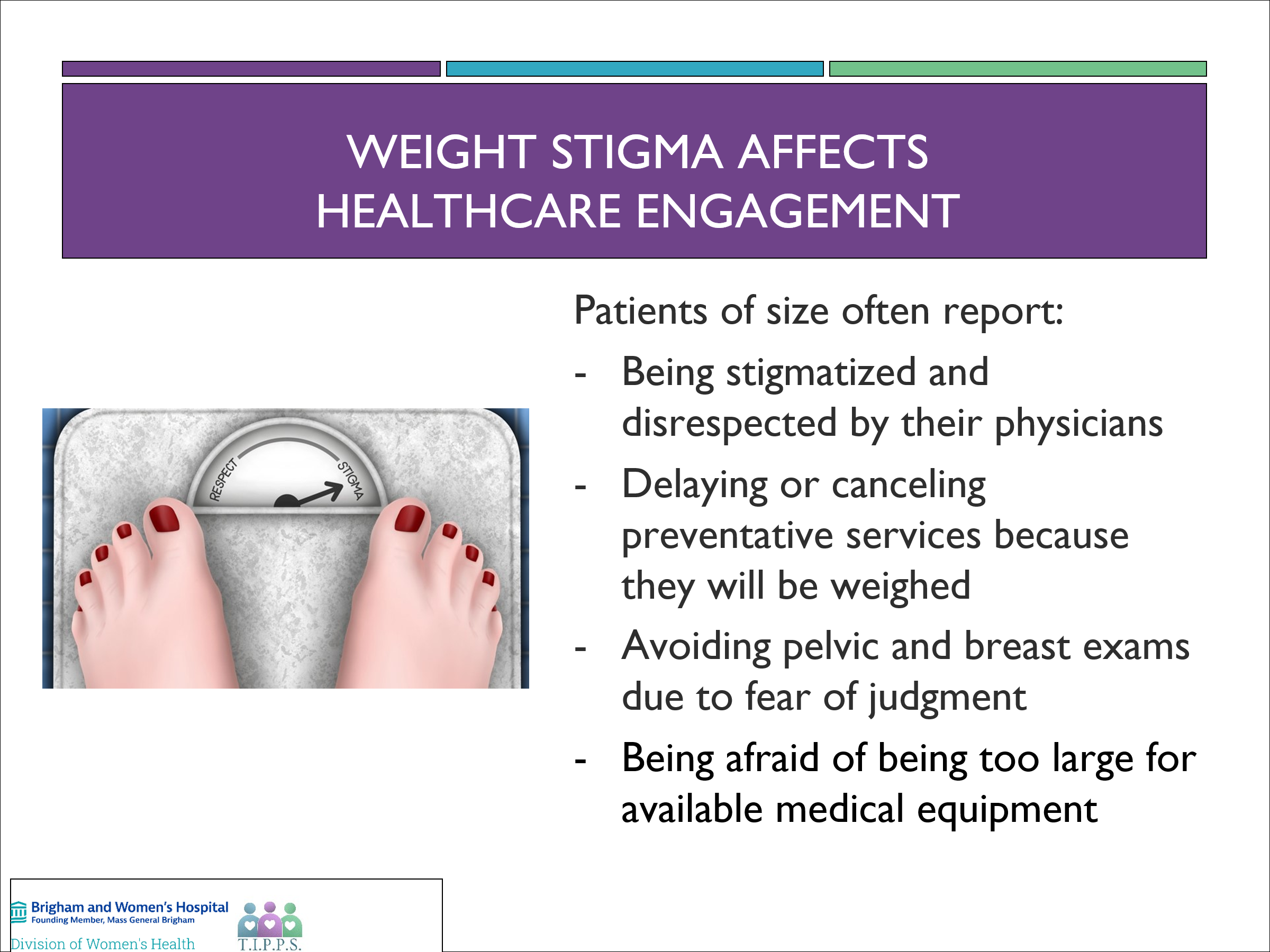


Figure S.8 Example of course content considering how weight stigma affects healthcare

Course script: Weight stigma affects healthcare engagement. In one study, 21% of participants reported they would look for a new doctor if they perceived stigmatization about their weight. Another showed that over 60% of patients complained that their physicians did not truly understand how difficult it was to be overweight. In that study, 24% of patients reported that their primary care practitioners sometimes did not believe them when they told them they do not eat that much.

(Puhl et al. Int J Obesity 2013; Wadden et al. Arch Family Med 2000)

Section B. Content from the track for clinicians


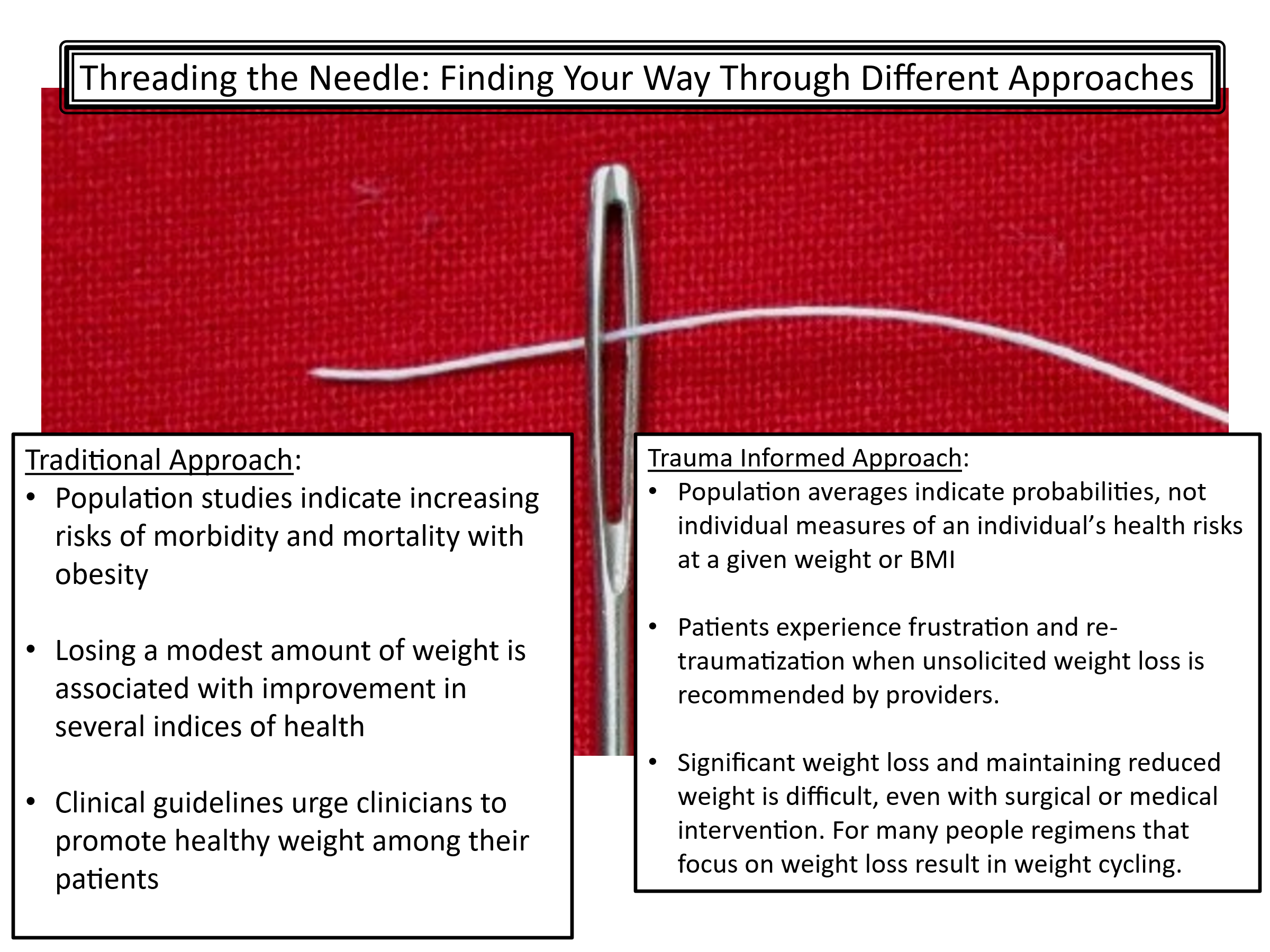


Figure S.9 Example of course content considering how weight stigma affects healthcare


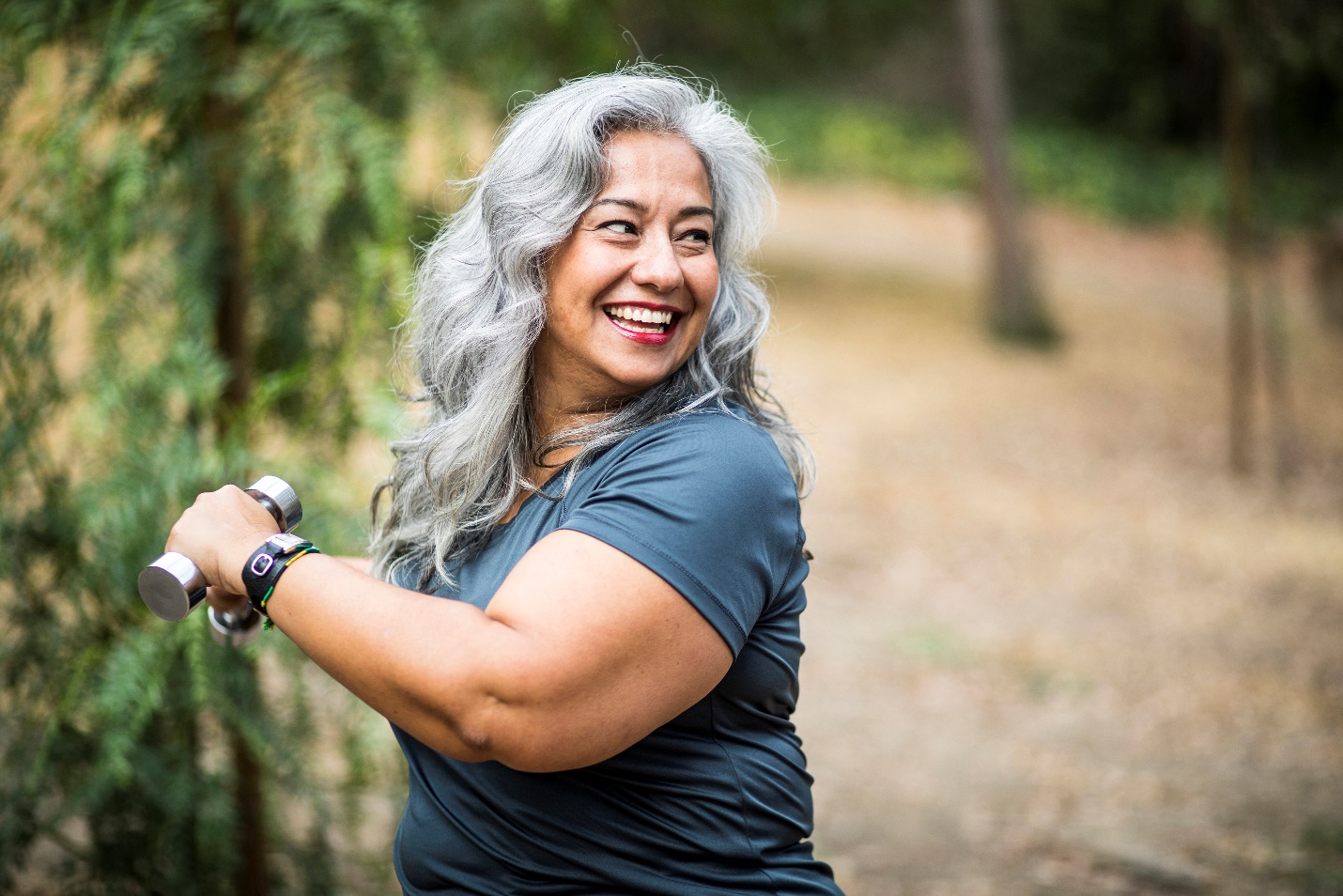


Figure S.10 Example of course content introducing alternatives to a focus on weight loss

Course script: Instead of a futile exercise of chasing a number on a scale, you could focus instead on encouraging your patients to increase their physical activity in ways that are sustainable and fit into their life and routines.


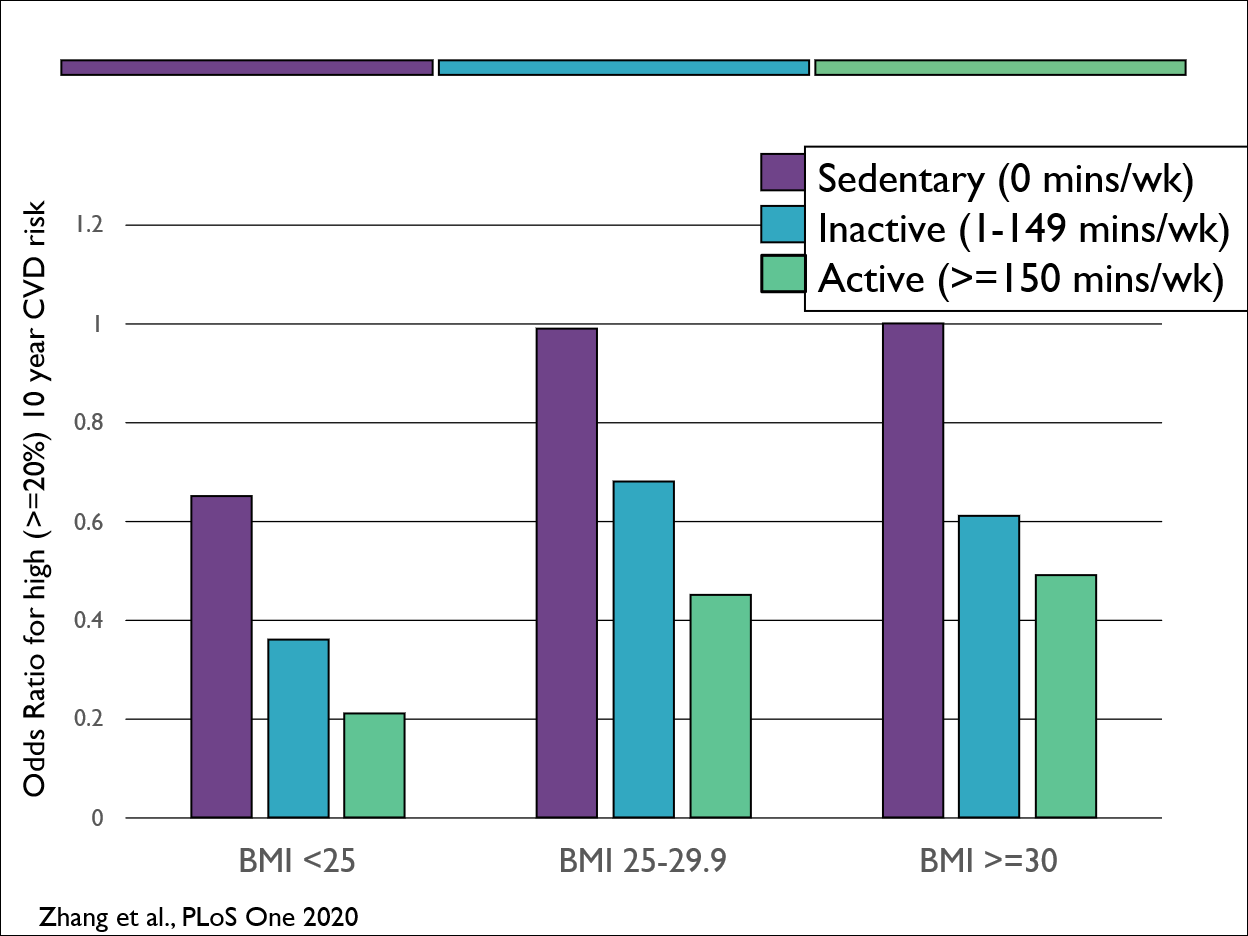


Figure S.11 Example of course content highlighting merits of weight loss alternatives

Course script: **More physical activity is associated with lower CVD risk ​for all body sizes**

Increased physical activity is associated with reduction in cardiovascular risk for all body sizes. Here, you can see in the National Health and Nutrition Examination Survey that, regardless of body size, moving from a sedentary lifestyle to one that includes up to 2.5 hours of physical activity per week meant a 30%-40% drop in the odds of having a high CVD risk score. Exercising 2.5 hours or more a week reaped further benefits, but the biggest change is in that first increase from none to some activity. The study authors noted that, ​“Physical activity was associated with a larger magnitude of reduced odds of 10-year CVD risk than weight status.”


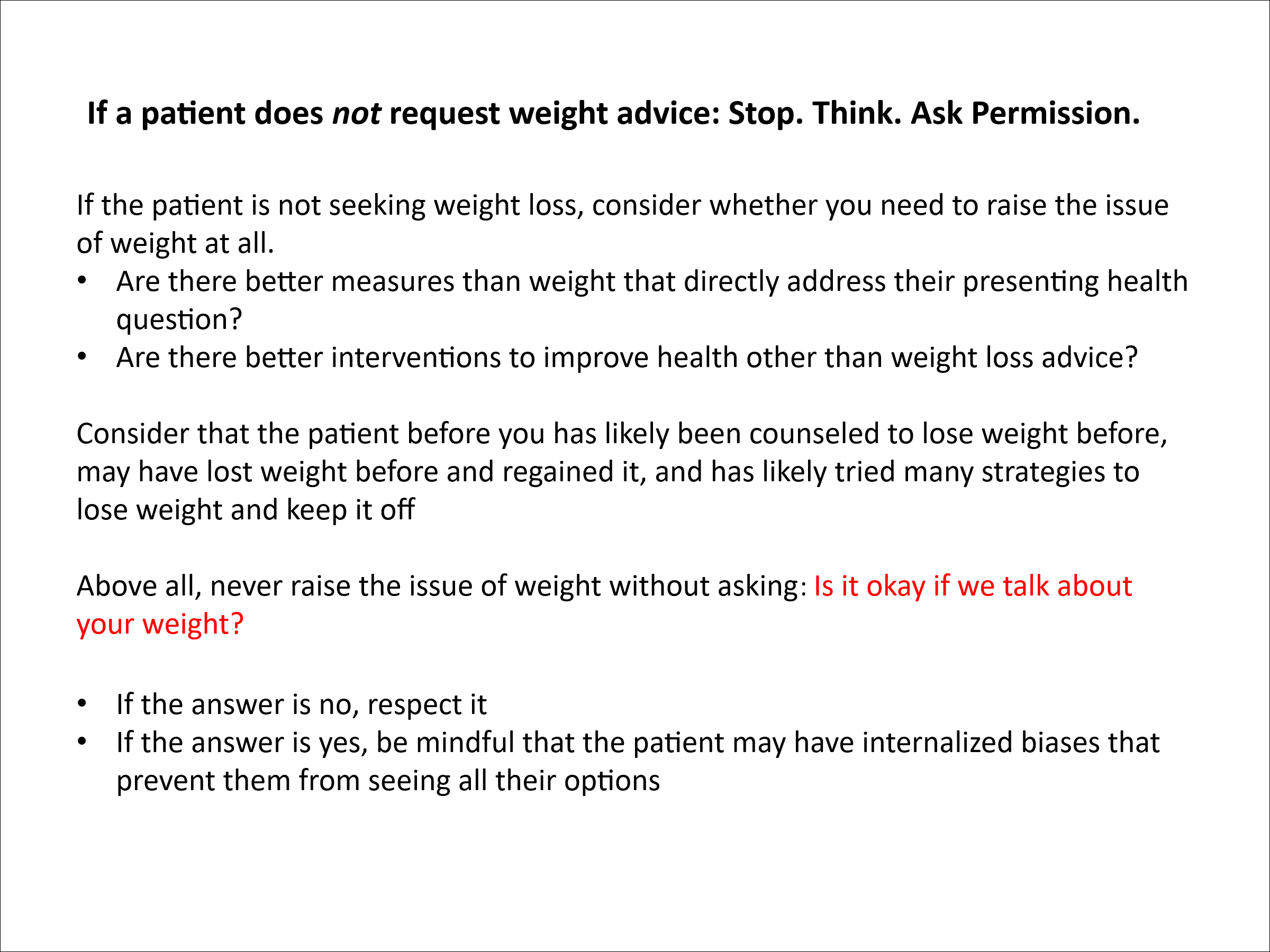


Figure S.12 Example of course content reminding clinicians to respect patient concerns


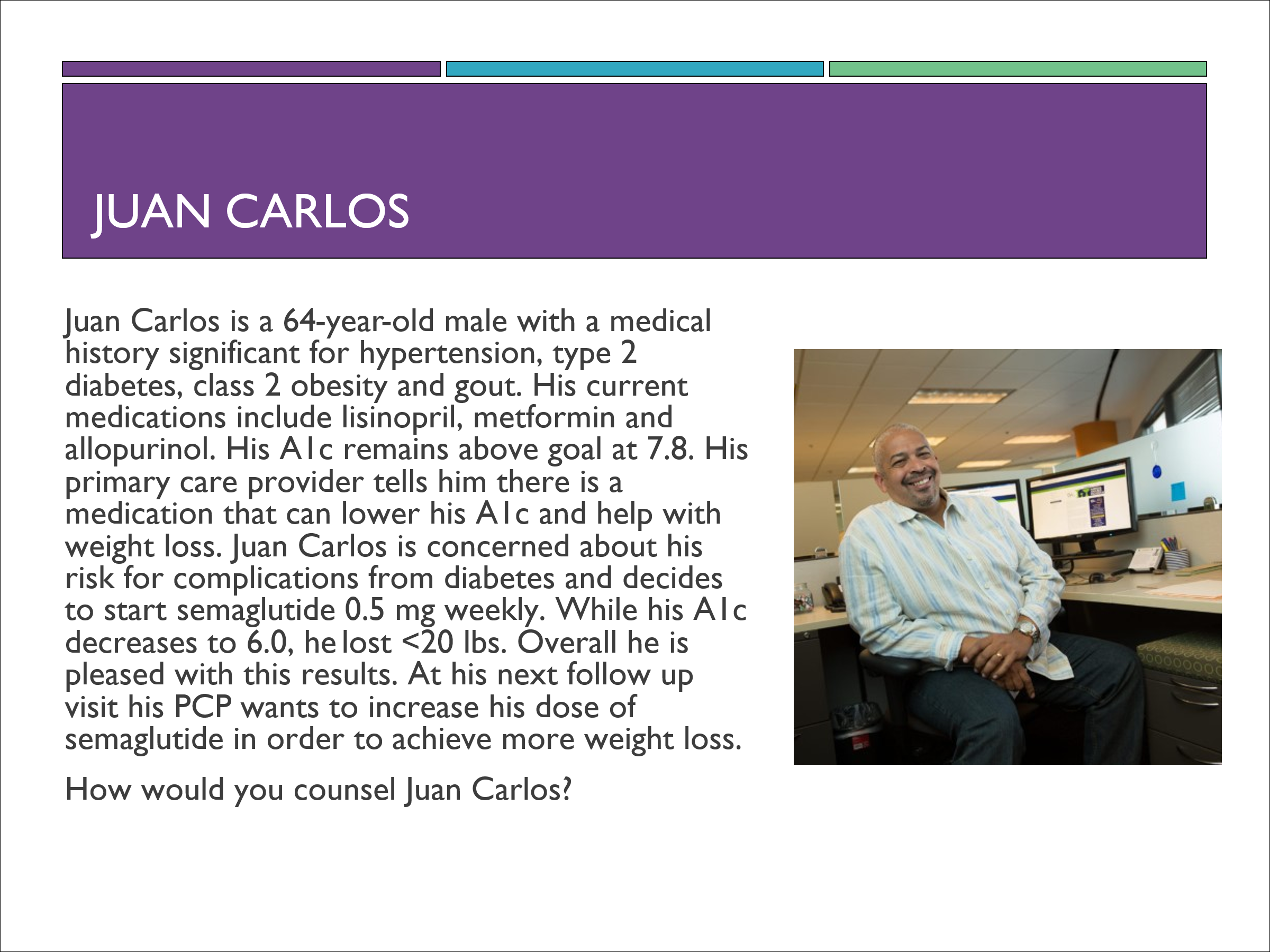


Figure S.13 Example of clinical case study

Course script: Patients need to feel they are part of their own treatment plan. If the patient feels they have reached their goal their wishes should be honored and respected. Juan Carolos should be congratulated. Consider how you would counsel a similar patient in a smaller body. All patients should be given the option to take the smallest dose of GLP medications that are effective for their health goals. Instead of focusing on weight loss, consider what risks Juan Carlos is most concerned about and see if there are other strategies to mitigate his risk.


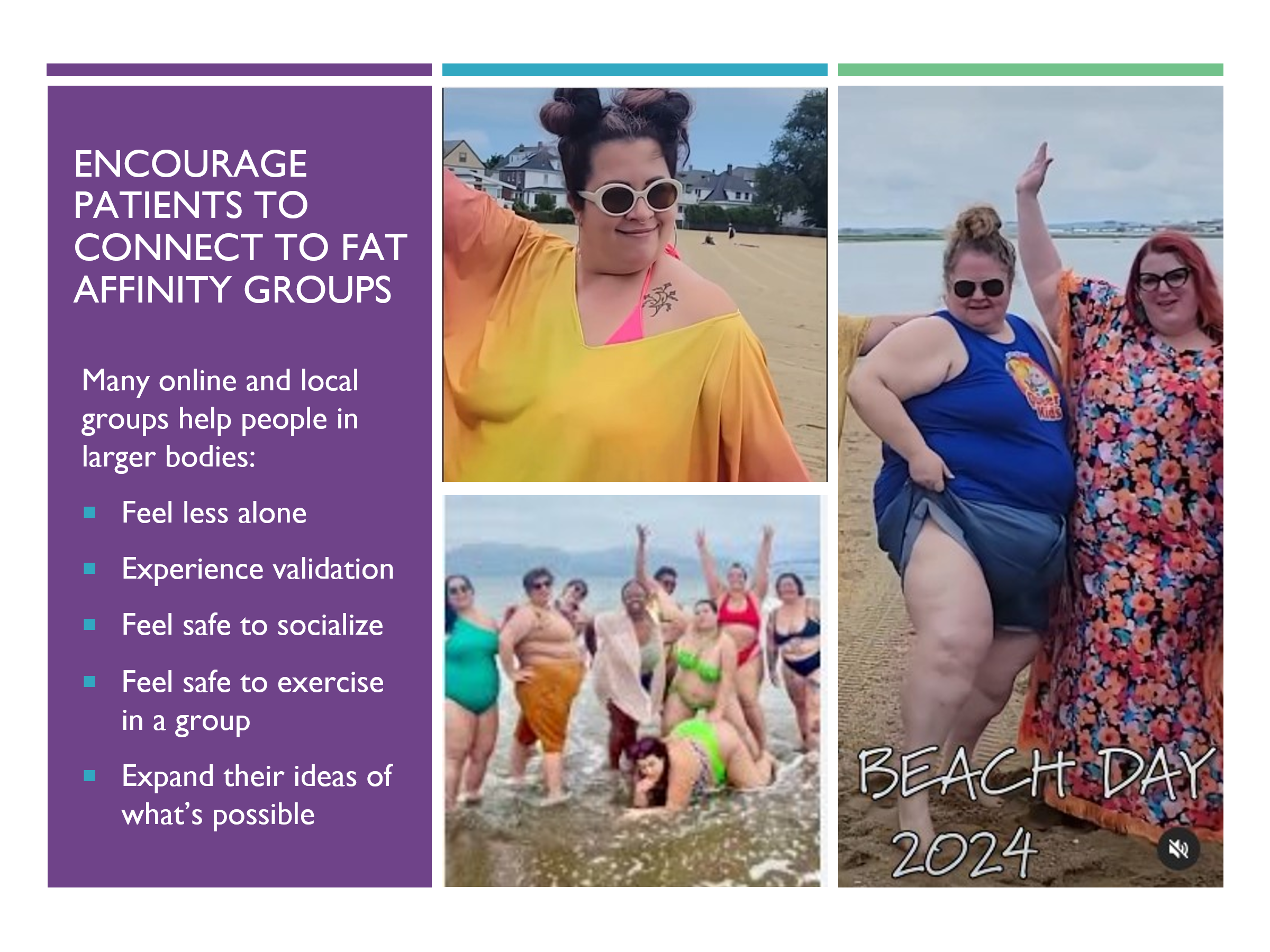
Figure S.14 Example of material promoting use of allyship resources


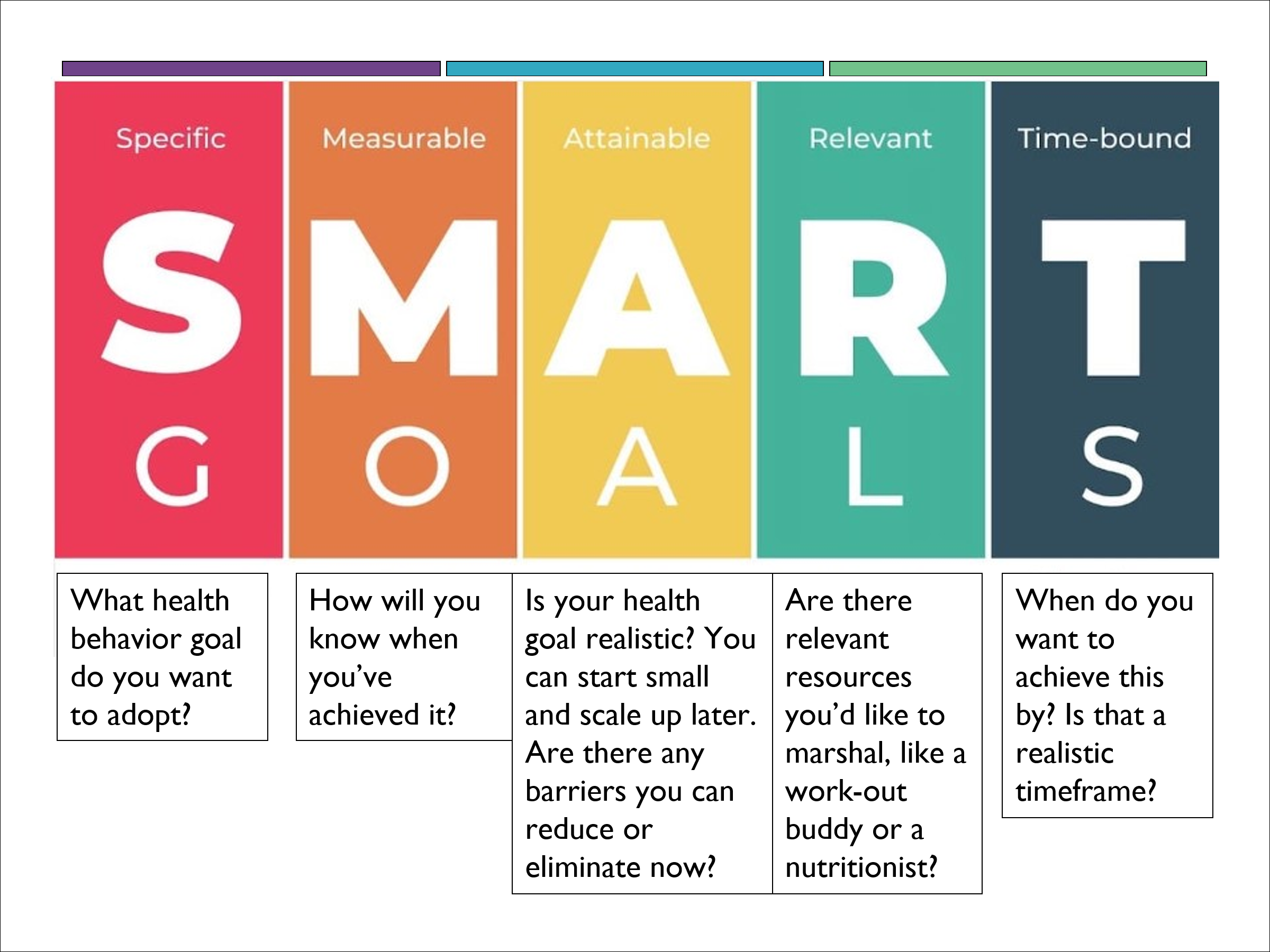


Figure S.15 Example of material promoting mutuality in clinical counseling

Course script: Your role is to help the patient define what a successful health outcome looks like for them using problem-solving techniques like the SMART goals principles outlined here. Focus on goals that matter to the patient, not just weight, size and appearance. You can open this conversation by asking “What does health success look like to you? What specific health behavior do you want to adopt?” You can help their chances of success by identifying a measurable goal and discussing whether it’s realistic. Starting small is often better. Discuss barriers that they know of and identify resources or referrals they might need. You can focus on a patient’s strengths. Finally, ask what they want the members of their healthcare team to know about them and their plan. This process will help the patient keep their plan realistic; you are teaching self-advocacy as you do this, which is motivating and empowering.

Section C. Content from the track for practice assistants


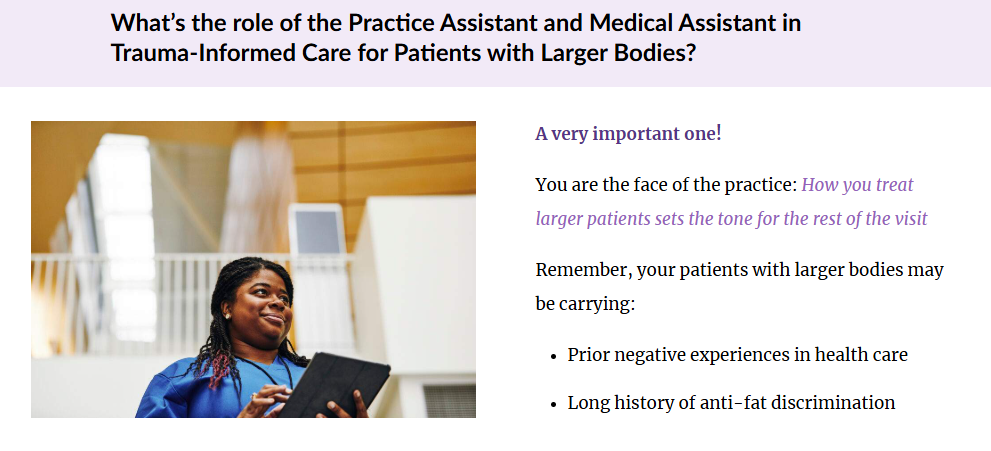


Figure S.16 Example from the introduction to the Practice Assistant track


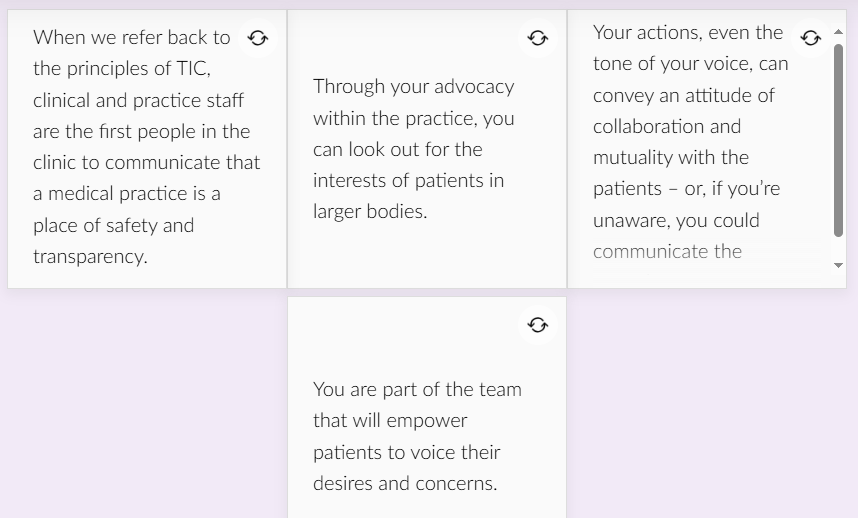


Figure S.17 Example of course material linking TIC principles to Practice Assistant role

Clinical and practice staff are invaluable members of the healthcare team. Even the most subtle change in your language or demeanor when you interact with a patient, particularly those in larger bodies, can begin to change the practice.

What you say and what you do makes an enormous difference!

Everyone in the practice contributes to the ‘hidden curriculum,’ the systemic biases that affect the care of patients. We all learn and unlearn these unspoken lessons from each other by example, regardless of where we sit in the hierarchy of the clinic. By putting dignity and respect first, you will help both your patients, your colleagues, and begin to create a more humane and equitable world. By educating yourself on the impact of weight bias and stigma on patient outcomes you can pave the way for the rest of the team to reduce future harm.


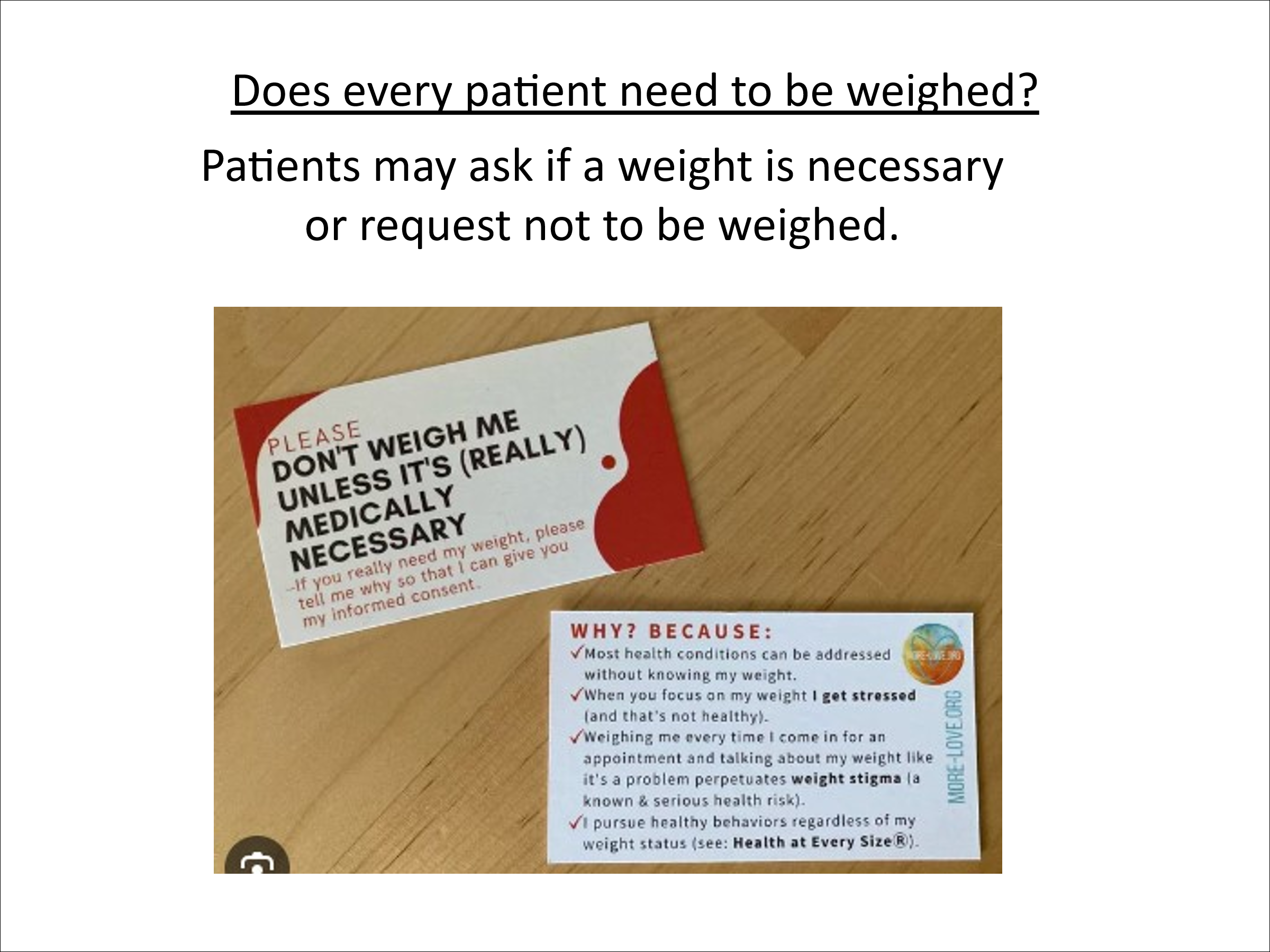


Figure S.18 Example of course material proposing alternatives to weighing at every visit

Course script: Increasingly, medicine is considering whether a weight needs to be recorded for every patient at *every* visit. There are lots of valid reasons why patients may not want to be weighed as well as reasons a patient *should* not be weighed. Regardless of body size, many patients find the process of being weighed uncomfortable or a violation of their privacy. For some, it can trigger feelings of shame stemming from childhood or worse by triggering a relapse of an eating disorder.  When possible, consider if the downside of being weighed is worth having the number in the chart.

**How Welcoming is Your Exam Room?**


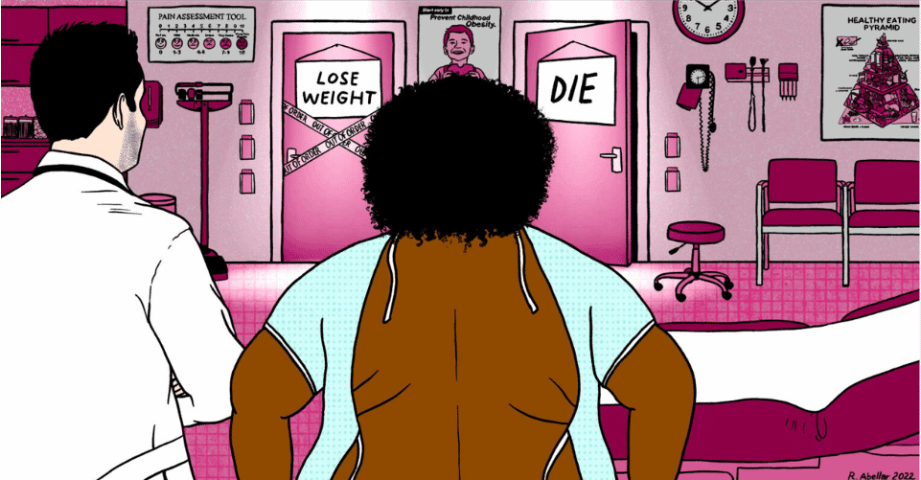


Figure S.19 Example of course material promoting a healthy clinic environment

Course script: What about images in your exam rooms? Even a nutrition poster may make a patient wary that a clinician will make assumptions and judgments about their diet. This is especially true for people who have experienced weight bias in the clinic or who have had eating disorders.
